# Tumor‐Targeted FABP5/STING Cascade Promote Radiofrequency Ablation Induced Ferroptosis and Intratumoral Immune Rewiring in Hepatocellular Carcinoma

**DOI:** 10.1002/advs.202507864

**Published:** 2025-09-16

**Authors:** Bufu Tang, Xiaojie Zhang, Yiting Sun, Jinhua Luo, Qiaoyou Weng, Cong Zhang, Zilin Wang, Shiji Fang, Yangrui Xiao, Liyun Zheng, Jianfei Tu, Rongfang Qiu, Yang Yang, Zhongwei Zhao, Weiqian Chen, Minjiang Chen, Jiansong Ji

**Affiliations:** ^1^ Zhejiang Key Laboratory of Imaging and Interventional Medicine Zhejiang Engineering Research Csaenter of Interventional Medicine Engineering and Biotechnology The Fifth Affiliated Hospital of Wenzhou Medical University Lishui Zhejiang 323000 China; ^2^ Key Laboratory of Precision Medicine of Lishui City Department of Radiology Lishui Central Hospital Lishui 323000 China; ^3^ Department of Radiology Lishui Hospital School of Medcine Zhejiang University Hangzhou 310000 China; ^4^ China Medical University Shenyang 110122 China; ^5^ Department of Interventional Radiology Zhongshan Hospital Fudan University Shanghai 200000 China

**Keywords:** FABP5, ferroptosis, HCC, RFA, STING

## Abstract

Radiofrequency ablation (RFA) has emerged as a critical therapeutic modality for hepatocellular carcinoma (HCC). Biomimetic magnetic nanoparticles further augment RFA efficacy by enhancing targeting precision and biocompatibility. This study introduces a novel nanocarrier co‐delivery system aimed at key molecular targets in HCC to amplify ferroptosis and reprogram intratumoral immunity, thereby improving the therapeutic outcomes of RFA. The proposed nanocarrier integrates cyclic arginine‐glycine‐aspartic acid (cRGD) and red blood cell membrane (RBCM), encapsulating superparamagnetic iron oxide (SPIO) to specifically target fatty acid‐binding protein 5 (FABP5) in tumor cells. Leveraging the superparamagnetic properties of SPIO, these nanoparticles enable real‐time monitoring and tracking through MRI. In vitro heat treatment of HCC cells simulates the RFA environment, while experiments employing the ferroptosis inhibitor Lipro1 conclusively demonstrate that the nanocarrier exerts anti‐tumor effects primarily through ferroptosis induction. Additionally, the impact on immunotherapy is underscored by combining the nanocarrier with anti‐PD‐L1 monoclonal antibodies. FABP5 overexpression in HCC tissues is strongly linked to RFA response. Targeting FABP5 using co‐delivery nanocarriers significantly enhances RFA efficacy. Furthermore, FABP5 deletion potentiates RFA‐induced ferroptosis and bolsters anti‐tumor immune responses, characterized by increased infiltration of CD8+ T cells and effector memory T cells, contributing to pronounced systemic anti‐tumor effects. Mechanistically, FABP5 inhibition activates the STING/TBK1 signaling pathway and modulates TBK1 protein stability. Notably, the nanocarrier system targeting FABP5 elevates PD‐L1 expression, and the combination of RFA with anti‐PD‐L1 therapy demonstrates synergistic efficacy against HCC. In conclusion, the FABP5‐targeting nanocarrier co‐delivery system offers a promising strategy to enhance RFA effectiveness in HCC, providing a novel framework for advancing clinical treatment approaches.

## Graphical abstract

Targeted FABP5 co‐delivery nanoparticles amplify RFA‐induced ferroptosis and intratumoral immune rewiring via STING/TBK1 pathway activation in HCC. The pH‐responsive lipid nanoshell was integrated with cyclic RGD peptide (cRGD) and red blood cell membrane (RBCM), and encapsulated with superparamagnetic iron oxide (SPIO) and sgFABP5 to construct FS@RBCM/cRGD‐phLips nanoparticles.FABP5 inhibition enhances iron‐dependent lipid peroxidation and promotes immunogenic cell death, leading to increased CD8+ T cell infiltration. The combination of FABP5‐targeted nanotherapy, RFA, and anti‐PD‐L1 synergistically suppresses tumor progression and metastasis, highlighting FABP5 as a promising diagnostic marker and therapeutic target in hepatocellular carcinoma.

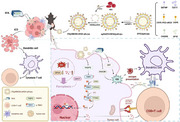

## 1 Introduction

Primary liver cancer, predominantly hepatocellular carcinoma (HCC), remains the fourth leading cause of cancer‐related mortality worldwide.^[^ ^1^ ^]^ The standard therapeutic options for HCC encompass surgical resection, liver transplantation, local ablative therapies, and radiofrequency ablation. However, the majority of HCC patients present with ineligibility for curative surgical intervention at diagnosis due to compromised hepatic functional reserve and advanced disease stage.^[^ ^2^, ^3^ ^]^ This clinical scenario underscores the critical need to develop and validate minimally invasive therapeutic approaches for managing patients with advanced HCC who are not candidates for surgical intervention.

Radiofrequency ablation (RFA) has emerged as a widely adopted curative treatment modality for early‐stage HCC, demonstrating a favorable safety profile and minimally invasive characteristics^[^ ^4^ ^]^ while enabling selective tumor ablation and optimally preserving healthy liver parenchyma, thus minimizing the risk of post‐procedural hepatic dysfunction.^[^ ^5^, ^6^ ^]^ Superparamagnetic iron oxide (SPIO) nanoparticles, characterized by their exceptional biocompatibility and superior MRI signal sensitivity, facilitate precise detection and tracking, thereby serving as effective T2‐weighted contrast agents for cancer diagnostics^[^ ^7^, ^8^ ^]^ Magnetic resonance imaging (MRI) serves as an essential imaging modality in RFA, offering superior soft tissue contrast for comprehensive tumor management, including pre‐procedural planning, real‐time guidance, and post‐ablation assessment. In this context, SPIO function as crucial contrast agents for precise tumor targeting and thermal ablation monitoring.^[^ ^9^, ^10^, ^11^ ^]^ SPIO‐enhanced MRI plays a fundamental role in tumor delineation, real‐time ablation monitoring, and post‐procedural assessment of residual lesions. Consequently, the engineering of SPIO nanoparticles constitutes a critical advancement in enhancing MRI‐guided RFA efficacy for HCC treatment. Nevertheless, SPIO nanoparticles in isolation provide solely diagnostic imaging capabilities without direct therapeutic efficacy.^[^ ^12^ ^]^ Incomplete ablation during HCC radiofrequency therapy frequently results in tumor progression and local recurrence, representing a significant therapeutic challenge. This critical unmet clinical need necessitates the development of dual‐functional contrast agents that simultaneously integrate real‐time imaging capabilities with therapeutic enhancement, representing a transformative strategy for improving treatment outcomes.

RFA has established itself as a pivotal therapeutic modality in the management of HCC. The therapeutic mechanism involves the placement of electrodes within tumor tissue to generate rapid thermal elevation, inducing coagulative necrosis of malignant cells.^[^ ^13^ ^]^ Moreover, RFA potentiates tumor cytotoxicity through the liberation of tumor antigens and subsequent activation of dendritic cells, thereby initiating an anti‐tumor immune response.^[^ ^14^ ^]^ Previous investigations have established that both STAT3 and METTL1 significantly influence the therapeutic efficacy of RFA in cancer treatment.^[^ ^15^, ^16^ ^]^ Our research demonstrates that FABP5 inhibition markedly enhances RFA efficacy in HCC by inducing immunogenic cell death and reprogramming the immunosuppressive tumor microenvironment toward a pro‐inflammatory phenotype.

Fatty acid‐binding protein 5 (FABP5) serves as an intracellular fatty acid chaperone protein, orchestrating lipid metabolism and cellular proliferation.^[^ ^17^, ^18^ ^]^ FABP5 is critically involved in the pathogenesis of numerous diseases, particularly cancer, wherein it facilitates the activation of transcription factors that promote tumorigenesis‐associated protein expression.^[^ ^19^ ^]^ In HCC, FABP5 exhibits significant upregulation, promoting tumor progression and metastatic dissemination.^[^ ^14^, ^20^ ^]^ Previous investigations employing sgRNA‐mediated FABP5 knockdown have demonstrated robust antitumor efficacy in both in vitro models and subcutaneous xenograft assays, validating its therapeutic potential in HCC.^[^ ^14^, ^21^, ^22^ ^]^ However, the systemic administration of unprotected sgRNA in vivo is constrained by rapid enzymatic degradation, inadequate tumor targeting specificity, and limited cellular internalization, collectively impeding sustained and precise FABP5 gene editing in malignant hepatocytes and restricting its therapeutic potential.^[^ ^23^, ^24^ ^]^ The development of safe and tumor‐targeted delivery systems for the concurrent delivery of FABP5‐editing sgRNA and SPIO nanoparticles represents a crucial strategy for advancing precision RFA in HCC treatment.

Lipid‐based nanomaterials, specifically those structured with phospholipid bilayers, have emerged as promising delivery platforms, exhibiting exceptional biocompatibility, capacity for co‐encapsulation of multiple therapeutic agents, and controlled drug release capabilities.^[^ ^25^, ^26^ ^]^ Several liposomal formulations, including paclitaxel and irinotecan, have received U.S. Food and Drug Administration approval and demonstrated sustained antitumor efficacy in clinical oncology.^[^ ^27^, ^28^ ^]^ Previous investigations have demonstrated that liposomes effectively encapsulate SPIO for diagnostic applications, while cationic liposomes serve as highly efficient delivery vectors for nucleic acid therapeutics, including siRNA and sgRNA.^[^ ^29^, ^30^ ^]^ Leveraging these advances, we developed tumor‐targeting liposomal formulations that simultaneously deliver SPIO and sgFABP5, enabling CRISPR‐mediated gene silencing concurrent with real‐time MRI monitoring of ablation efficacy in HCC. This integrated delivery system, which facilitates simultaneous imaging guidance and local therapeutic enhancement, represents a clinically viable strategy for optimizing post‐ablation outcomes in HCC treatment. To elucidate the anti‐tumor efficacy of targeting tumor cell‐intrinsic FABP5 co‐delivery nanocarriers combined with RFA treatment in HCC, nanoparticles were categorized into four groups based on their composition in subsequent experiments: RP:@RBCM/cRGD‐phLips (control group), SRP:SPIO@RBCM/cRGD‐phLips, FRP:sgFABP5@RBCM/cRGD‐phLips, and FSRP:FS@RBCM/cRGD‐phLips. These groups represented distinct therapeutic strategies for HCC.

## 2 Result

### 2.1 FABP5 is Highly Expressed in Hepatocellular Carcinoma Tissues and is Correlated with RFA

**Figure 1:**
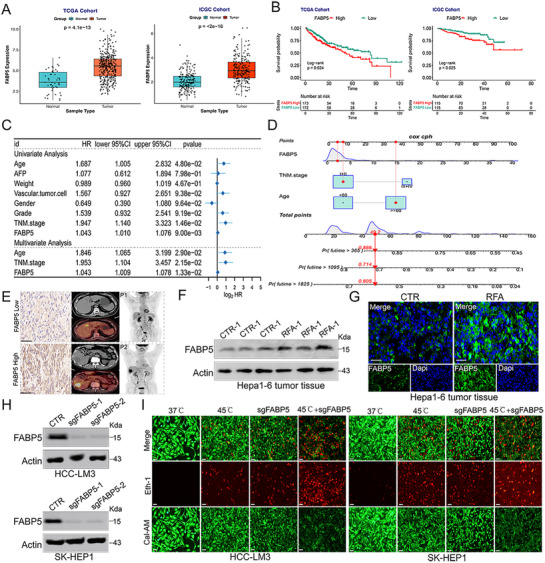
Upregulation of FABP5 expression in hepatocellular carcinoma tissues and its correlation with RFA A) Box plots illustrating FABP5 expression levels in normal versus tumor samples. B) Survival analysis comparing the prognosis of patients with HCC exhibiting high and low FABP5 expression in the TCGA‐LIHC and ICGC cohorts. C) Forest plot identifying FABP5 as an independent risk factor for HCC. D) Nomogram for calculating individualized risk scores. E) Imaging studies demonstrating the association between FABP5 expression and tumor progression. F) Western blot analysis revealing changes in FABP5 expression following RFA treatment. G) Immunofluorescence assays indicating FABP5 upregulation in tumor tissues post‐RFA. H) Western blot analysis confirming the knockdown efficiency of plasmids targeting FABP5. I) Cell viability of HCC‐LM3 and SK‐HEP‐1 cell lines assessed using calcein AM (green) and ethidium homodimer‐1 (red) dual staining under specified treatments (n = 3). Scale bar = 100 µm.

Data from the TCGA‐LIHC, ICGC‐LIRI‐JP, and GEO cohorts were analyzed to compare FABP5 expression levels between HCC lesions and corresponding normal liver tissues. FABP5 expression was significantly higher in tumor tissues than in normal tissues (**Figures** **1**A; S1A, Supporting Information). Elevated FABP5 expression was strongly associated with reduced survival rates in patients with HCC (Figures 1B; S1B, Supporting Information). Univariate and multivariate Cox regression analyses confirmed that FABP5 expression serves as an independent risk factor for poor prognosis, and a nomogram incorporating these risk factors was constructed to calculate individual risk scores (Figure 1C,D). Additionally, patients with HCC exhibiting high FABP5 expression demonstrated a higher degree of malignant progression (Figure 1E), implicating FABP5 in tumor advancement.

Following RFA treatment, FABP5 expression was observed to increase significantly (Figures 1F,G; S1C, Supporting Information). These results suggest that reducing FABP5 expression could improve the therapeutic efficacy of RFA. To investigate this, a plasmid was constructed to achieve FABP5 knockout, and Western blot analysis confirmed successful knockout (Figure 1H). To model RFA conditions, HCC‐LM3 and SK‐HEP1 cells were subjected to heat treatment at 45 °C in vitro. Plasmids targeting FABP5 were introduced into HCC cells to further elucidate its role. The results demonstrated that both heat treatment and sgFABP5 delivery induced cell death, and the combination of these treatments significantly enhanced the cytotoxic effects (Figures 1I; S1D, Supporting Information).

### 2.2 Construction and Characteristics Evaluation of Targeting Tumor Cell‐Intrinsic FABP5 Co‐Delivery Nanocarrier

**Figure 2:**
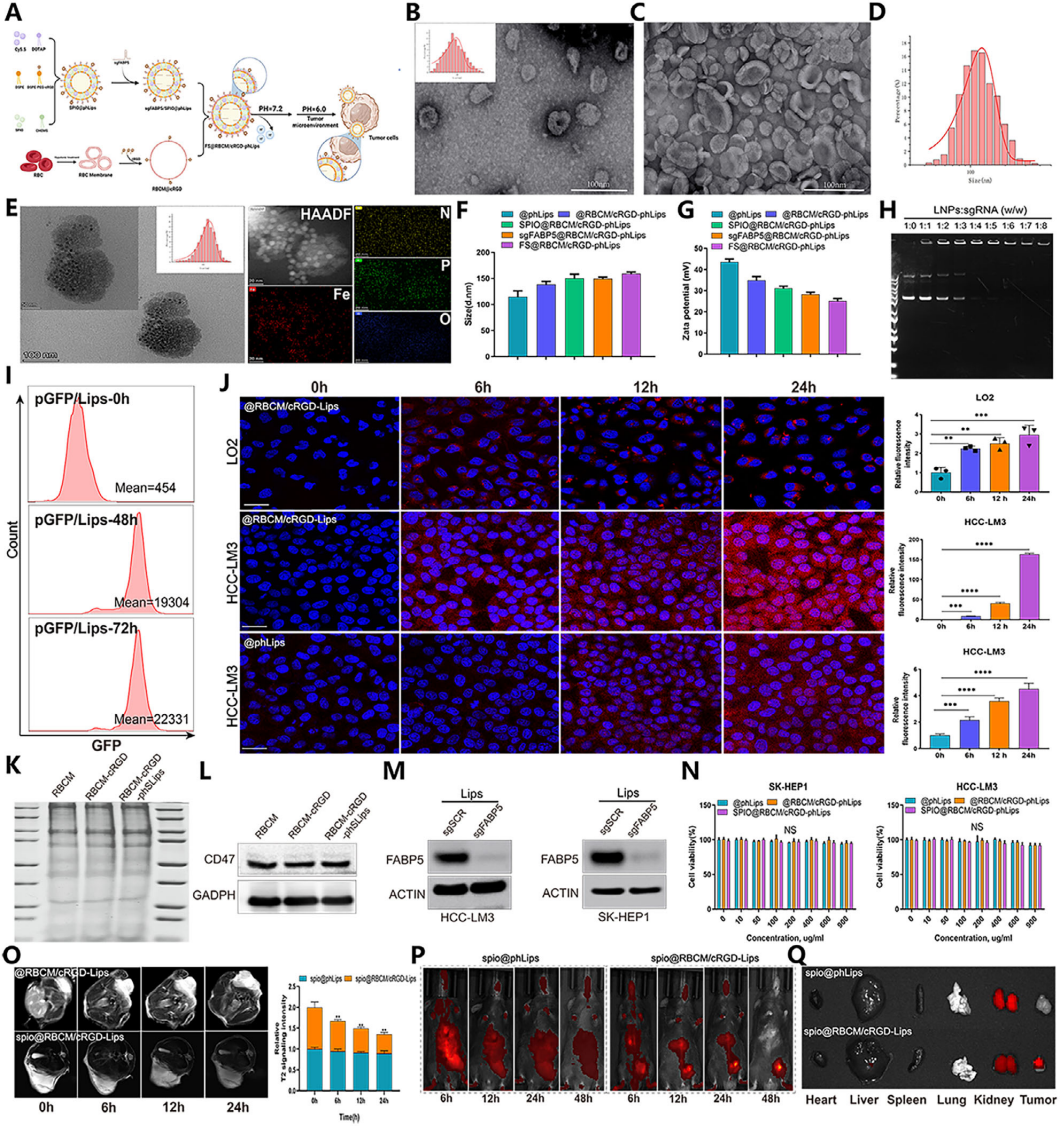
Development and characterization of FS@RBCM/cRGD‐phLips nanoparticles A) Schematic diagram depicting the structural modifications of lipid nanoparticles. B,C) Representative transmission electron microscopy (TEM) images displaying the morphology of the modified lipid nanoparticles. D) Dynamic light scattering (DLS) analysis demonstrating the size distribution of the nanoparticles. E) Elemental analysis of the nanoparticles was conducted using TEM. F) Statistical histograms presenting size measurements of five distinct LNP formulations. G) Zeta potential analysis comparing the surface charge of the formulations. H) Agarose gel electrophoresis evaluating the binding efficiency of LNP‐sgRNA complexes at varying mass ratios. I) Flow cytometry assessing the transfection efficiency of sgRNA‐loaded LNPs by quantifying GFP fluorescence intensity. J) Immunofluorescence micrographs and time‐course analysis illustrating mean fluorescence intensity (MFI) in LO2 and HCC‐LM3 cells. K) Coomassie blue staining comparing protein compositions in RBCM, RBCM‐cRGD conjugates, and dual‐targeted RBCM/cRGD‐phLips nanoparticles. L) Western blot analysis of CD47 expression in RBCM, RBCM‐cRGD, and RBCM/cRGD‐phLips formulations, using GAPDH as the internal control and CD47 as an erythroid marker. M) Western blot results indicating FABP5 expression in HCC‐LM3 and SK‐HEP‐1 cells treated with or without LNP‐encapsulated sgFABP5. N) Cell viability assays comparing the effects of @phLips, @RBCM/cRGD‐phLips, and SPIO@RBCM/cRGD‐phLips on SK‐HEP‐1 and HCC‐LM3 cells. O) Representative MRI scans illustrating HCC progression under different treatments, accompanied by quantitative analysis of relative T2‐weighted signal intensity for SPIO@phLips versus SPIO@RBCM/cRGD‐phLips. P,Q) Fluorescence imaging of in vivo and ex vivo distributions of nanoparticles in organs and tumors.

Although FABP5 knockout significantly enhances the anti‐tumor efficacy of RFA, the in vivo instability of the sgFABP5 plasmid limits its clinical applicability. To address this, a tumor cell‐intrinsic FABP5‐targeting co‐delivery system was developed to enhance therapeutic efficacy specifically (**Figure** **2**A). The morphological characteristics of the nanoparticle suspension are shown in Figure S2 (Supporting Information), with TEM imaging revealing the structure of @RBCM/cRGD‐phLips (Figure 2B,C). The nanoparticles demonstrated a hydrodynamic diameter of 136 ± 28 nm (Figure 2D), and elemental analysis confirmed the presence of iron (Fe) on their surfaces (Figure 2E). Zeta potential measurements and particle size distributions of the engineered nanoparticles are presented in Figure 2F,G. Gel electrophoresis analysis indicated the successful incorporation of sgRNA into the LNPs at a 1:4 weight ratio (Figure 2H). Time‐course fluorescence analysis showed a significant increase in GFP intensity at 48 h, which further increased at 72 h after nanoparticle transfer into cells. Immunofluorescence (IF) staining confirmed successful sgRNA loading and enhanced nanoparticle uptake in HCC‐LM3 and SK‐HEP1 cells after 48 h (Figures 2I; S3, Supporting Information). A comparative targeting efficiency analysis revealed significantly higher uptake of @RBCM/cRGD‐phLips in HCC‐LM3 cells than in LO2 cells, underscoring its tumor‐targeting capabilities (Figure 2J). Coomassie blue staining demonstrated the stability and intactness of RBCM in the nanoparticles (Figure 2K). CD47, a key protein on the RBCM, interacts with the signal‐regulatory protein alpha (SIRPα) on phagocytes, inhibiting phagocytosis of @RBCM/cRGD‐phLips by immune cells. This mechanism significantly reduces macrophage‐mediated clearance, resulting in prolonged in vivo retention of the nanoparticles (Figure 2L). Delivery of sgFABP5‐loaded lipid nanoparticles (Lips) to HCC‐LM3 and SK‐HEP1 cells led to a marked reduction in FABP5 expression (Figure 2M). Cell viability assays using CCK‐8 showed negligible cytotoxic effects of the nanoparticles and SPIO‐containing nanoparticles on cell viability (Figure 2N). T2‐weighted imaging demonstrated strong tumor‐targeting properties of SPIO@RBCM/cRGD‐phLips, enabling effective nanoparticle detection and tracking (Figure 2O). In vivo and ex vivo fluorescence imaging confirmed preferential accumulation of SPIO@RBCM/cRGD‐phLips in tumor tissues compared to organs such as the heart and liver, further emphasizing the nanoparticles’ tumor‐specific targeting capabilities (Figures 2P,Q; S4, Supporting Information). These results validate the successful engineering of LNPs capable of stably and effectively delivering sgFABP5 and SPIO to target sites. Additionally, the strong MRI sensitivity and tumor‐targeting properties of these nanoparticles further enhance their potential for precise therapeutic applications.

### 2.3 Targeted FABP5 Co‐Delivery Nanoparticles Enhance RFA‐Mediated Ferroptosis in Hepatocellular Carcinoma Cells

**Figure 3:**
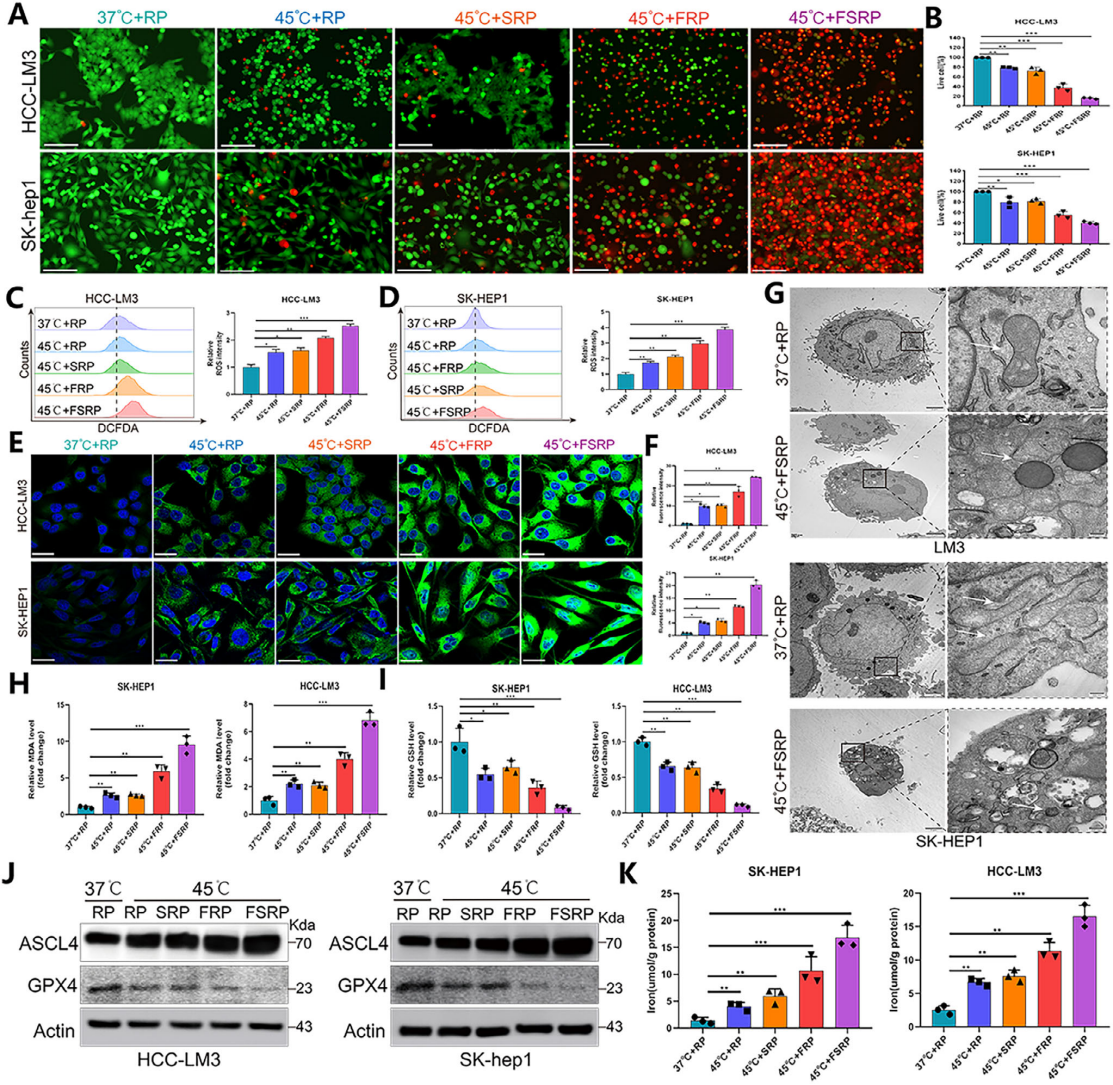
Targeted delivery of FABP5 nanoparticles enhances RFA‐mediated ferroptosis in hepatocellular carcinoma cells A) Cell viability of HCC‐LM3 and SK‐HEP‐1 cells evaluated under five treatment conditions using calcein AM (green, viable cells) and Eth‐1 (red, non‐viable cells) double staining. Scale bar = 100 µm. B) Percentage of viable cells for HCC‐LM3 and SK‐HEP‐1 under different treatment conditions. C,D) Flow cytometric analysis of reactive oxygen species (ROS) levels in HCC‐LM3 and SK‐HEP‐1 cells across five treatment groups, measured by DCFDA fluorescence intensity, with the 37 °C+RP group serving as the control. E) Representative immunofluorescence images showing C11‐BODIPY staining of HCC‐LM3 and SK‐HEP‐1 cells under the specified treatments. F) Quantitative analysis of relative mean fluorescence intensity (MFI) for HCC‐LM3 and SK‐HEP‐1 cells. G) Representative TEM images illustrating mitochondrial ultrastructural alterations in HCC‐LM3 and SK‐HEP‐1 cells following combined treatment with SPIO and sgFABP5‐loaded nanocarriers. H) Quantitative analysis of relative MDA levels in SK‐HEP‐1 and HCC‐LM3 cells. I) Quantitative analysis of relative GSH levels in SK‐HEP‐1 and HCC‐LM3 cells. J) Western blot analysis examining PCNA and GPX4 expression levels in HCC‐LM3 and SK‐HEP‐1 cells under the indicated treatments. K) Quantitative analysis of intracellular iron levels in SK‐HEP‐1 and HCC‐LM3 cells, with actin used as a loading control. Statistical significance, ^*^ *p*< 0.05; ^**^ *p*< 0.01; ^***^ *p*< 0.001.

To simulate RFA in vitro, HCC‐LM3 and SK‐HEP1 cells were subjected to heat treatment at 45 °C, followed by the introduction of the respective nanoparticle formulations into the tumor cells. Live/dead staining demonstrated that sgFABP5 delivery alone inhibited tumor cell proliferation, while the co‐delivery of SPIO and sgFABP5 significantly enhanced anti‐tumor effects, inducing higher levels of tumor cell death (**Figure** **3**A,B). Ferroptosis is a distinct form of cell death characterized by iron‐dependent phospholipid peroxidation, which is modulated by various cellular metabolic pathways. A decrease in glutathione peroxidase 4 (GPX4) levels, coupled with an accumulation of lipid‐reactive oxygen species (ROS), can trigger ferroptosis.^[^ ^31^ ^]^ Key features of ferroptosis include elevated iron ion concentrations, increased lipid peroxidation, and mitochondrial sequestration.^[^ ^32^ ^]^ Upon treatment with double‐loaded nanoparticles, a significant rise in ROS accumulation was observed in tumor cells, as evidenced by the DCFDA probe, which measures ROS intensity (Figure 3C,D). The C11‐BODIPY assay further confirmed increased lipid peroxidation in tumor cells (Figure 3E,F). TEM analysis revealed pronounced mitochondrial structural abnormalities, including wrinkling and cristae reduction, in cells subjected to RFA combined with double‐loaded nanoparticles (Figure 3G). Additionally, malondialdehyde (MDA) levels were significantly elevated, while glutathione (GSH) levels were reduced in these cells (Figure 3H,I). At the molecular level, GPX4 expression, a critical regulator of ferroptosis, was markedly decreased following treatment with RFA and double‐loaded nanoparticles (Figure 3J). Conversely, ASCL4 expression and intracellular iron content were significantly elevated, further supporting the induction of ferroptosis (Figure 3K). In summary, these results indicate that nanoparticles co‐delivering SPIO and sgFABP5 effectively induce tumor cell death and enhance anti‐tumor efficacy by promoting ferroptosis.

### 2.4 Nanocarrier‐Mediated FABP5 Knockout Promotes RFA‐Triggered Anti‐Tumor Immune Effects in HCC

**Figure 4:**
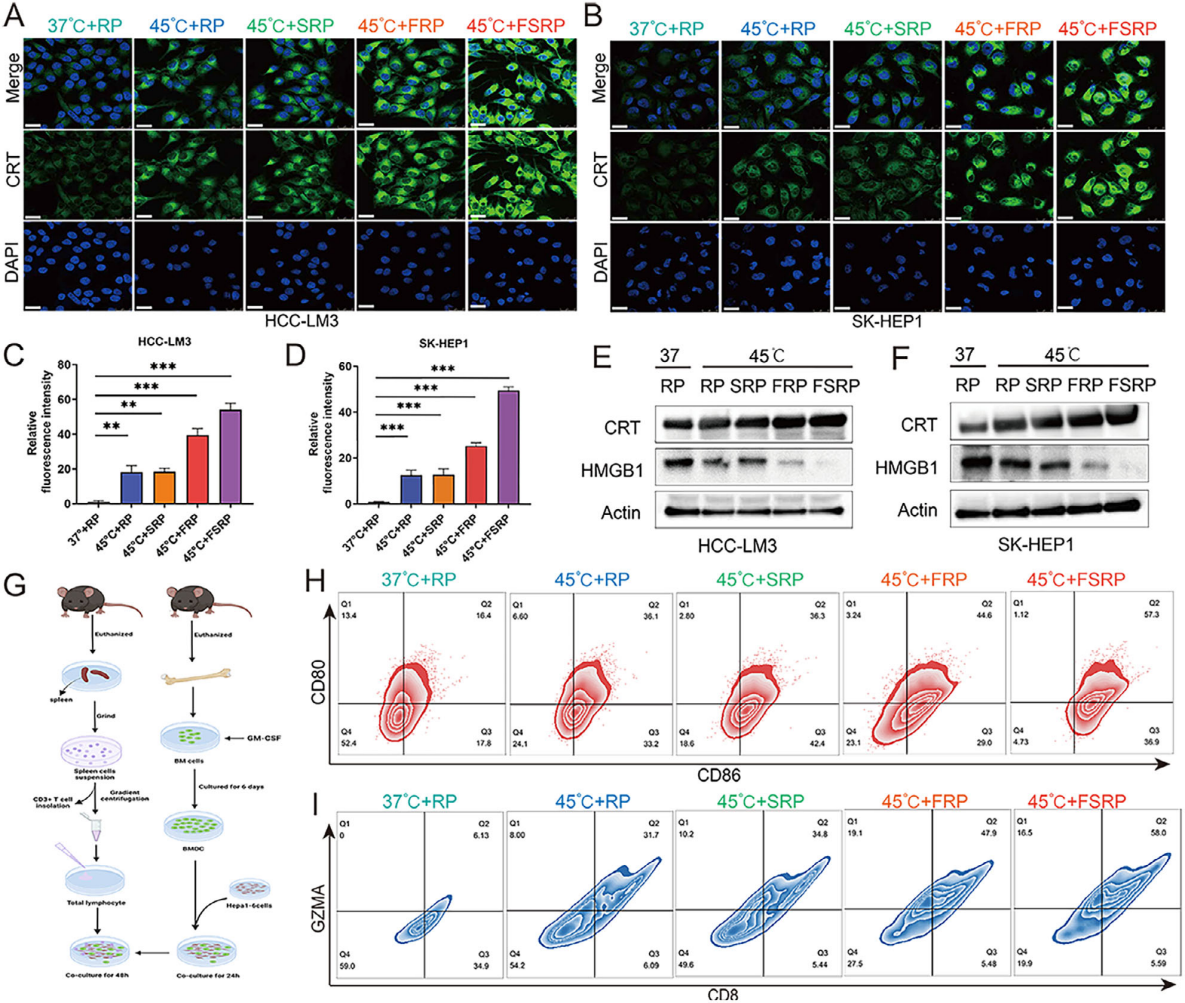
Targeted FABP5 co‐delivery system promotes RFA‐induced anti‐tumor immune effects in HCC A,B) Representative immunofluorescence images depicting calreticulin (CRT) expression under different treatment conditions. C,D) Quantitative analysis of CRT expression levels in HCC‐LM3 (C) and SK‐HEP‐1 (D) cells across treatment groups. E,F) Western blot analysis evaluating CRT and HMGB1 expression in HCC‐LM3 (E) and SK‐HEP‐1 (F) cells under the specified treatments, with actin used as a loading control. G) Schematic representation outlines the cell co‐culture and treatment protocol. H) Flow cytometry pseudocolor plot depicting CD80 and CD86 expression on BMDCs. I) Flow cytometry pseudocolor plot showing GZMA and CD8 expression on co‐cultured cells. Statistical significance, ^*^ *p*< 0.05; ^**^ *p*< 0.01; ^***^ *p*< 0.001.

Immune cell death (ICD) is a form of cancer cell death triggered by specific chemotherapeutic agents, oncolytic viruses, physicochemical therapies, photodynamic therapy, and radiotherapy.^[^ ^33^, ^34^ ^]^ In recent years, nanoparticle‐based therapeutic approaches have been increasingly utilized to induce and enhance ICD, thereby amplifying the efficacy of cancer immunotherapy.^[^ ^35^, ^36^ ^]^ While RFA can induce ICD, its effects are relatively transient and limited in intensity. Calreticulin (CRT) and High Mobility Group Box 1 (HMGB1) serve as key markers of ICD.^[^ ^37^ ^]^ IF staining revealed that CRT expression was markedly elevated when RFA was combined with double‐loaded nanoparticles (**Figure** **4**A–D). Additionally, ELISA assays demonstrated increased extracellular HMGB1 expression, corroborating these observations (Figure S5A,B, Supporting Information). Western blot analysis showed a significant upregulation of CRT expression alongside a reduction in HMGB1 levels in cells co‐treated with RFA and double‐loaded nanoparticles (Figure 4E,F). These results indicate that inhibiting FABP5 substantially enhances ICD and strengthens the anti‐tumor effects of RFA. To explore the impact of ICD on DC antigen‐presenting capacity, a co‐culture system of bone marrow dendritic cells (BMDCs) and Hepa1‐6 cells was established (Figure 4G). Hepa1‐6 cells were treated with FS@RBCM/cRGD‐phLips and RFA, followed by flow cytometry analysis. The results demonstrated a significantly higher percentage of CD80+CD86+ DCs, indicating increased DC maturation and antigen‐presenting capacity (Figures 4H; S5C, Supporting Information). Furthermore, a higher percentage of GZMA+CD8+ T cells was observed, signifying a robust enhancement in immune response activation (Figures 4I; S5D, Supporting Information). In summary, these results highlight that nanocarrier‐mediated FABP5 targeting significantly enhances RFA‐induced ICD and activates DC‐mediated immune responses, thereby amplifying anti‐tumor efficacy in HCC.

### 2.5 Targeted FABP5 Co‐Delivery Nanoparticles Activate the STING/TBK1 Pathway by Affecting TBK1 Protein Stability

**Figure 5:**
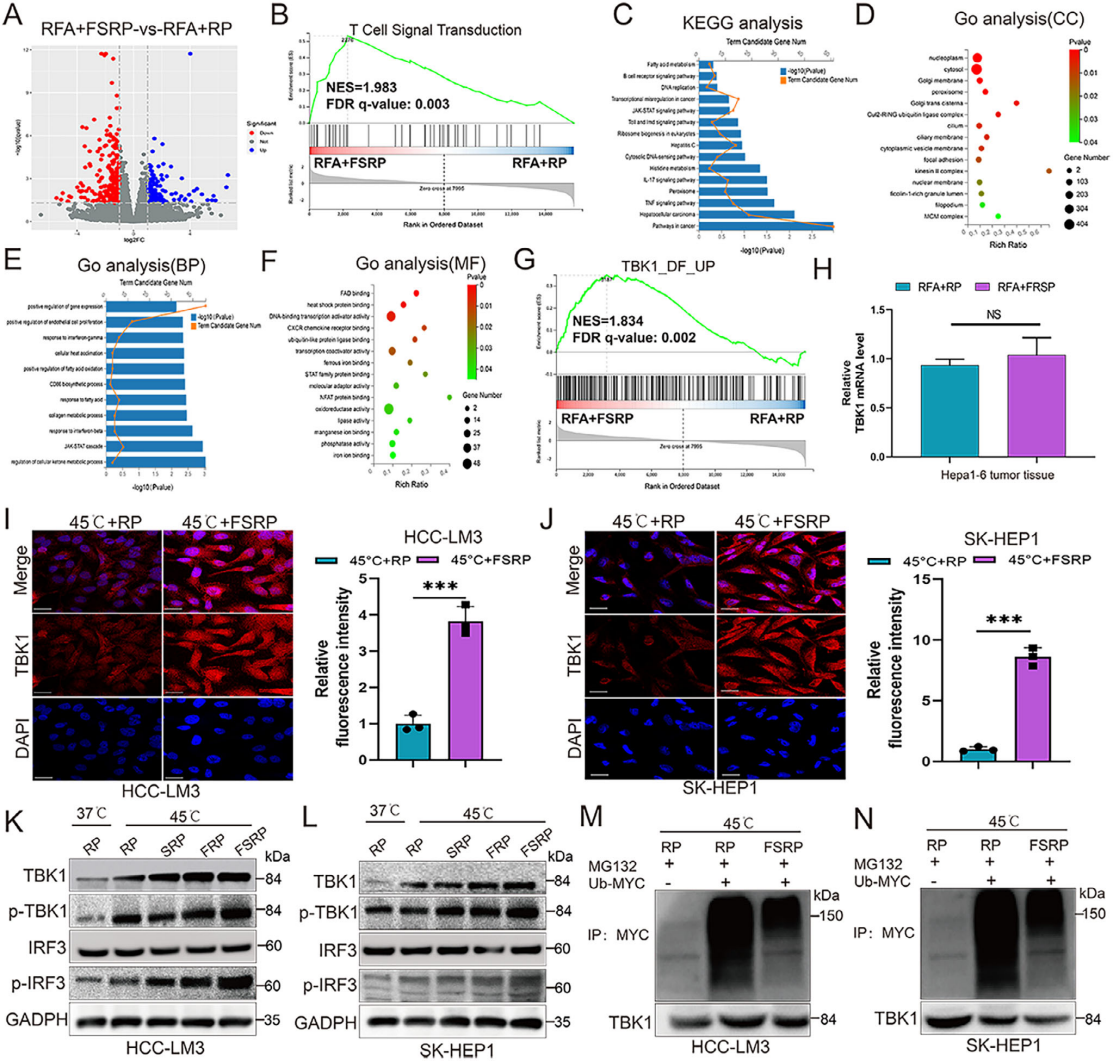
Nanocarrier‐mediated FABP5 knockout activates the STING/TBK1 pathway by modulating TBK1 protein stability. A) Volcano plot illustrating differentially expressed genes after RFA treatment with or without FSRP intervention. B) GSEA demonstrating that RFA combined with FSRP intervention is associated with T cell signaling pathway activation. C) KEGG pathway analysis highlighting signaling alterations in the tumor and immune microenvironment. D–F) GO analysis was performed from three aspects: cellular components (CC), biological processes (BP), and molecular functions (MF). G) GSEA revealing TBK1 pathway activation under RFA+FSRP treatment. H) Relative TBK1 mRNA expression levels quantified in Hepa1‐6 tumor tissues across treatment groups. I, J) Immunofluorescence staining showing TBK1 protein levels following 45 °C+FSRP treatment in HCC‐LM3 and SK‐HEP1 cells. K, L) Western blot analysis demonstrating the expression levels of TBK1, phosphorylated TBK1 (P‐TBK1), IRF3and p‐IRF3 in HCC‐LM3 (K) and SK‐HEP‐1 (L) cells under various conditions. M, N) Western blot analysis of ubiquitin (Ub) expression in HCC‐LM3 (M) and SK‐HEP1 (N) cells with or without sgFABP5 treatment after MG132 exposure. Statistical significance, ^*^ *p*< 0.05; ^**^ *p*< 0.01; ^***^ *p*< 0.001.

To further investigate the regulatory mechanisms underlying the combined effects of FABP5 knockout and RFA in HCC, transcriptome sequencing was performed on RFA+FSRP and RFA+RP‐treated LM3 cell samples. DEGs were identified and visualized using a volcano plot (**Figure** **5**A). Gene Set Enrichment Analysis (GSEA) of upregulated DEGs revealed that RFA+FSRP treatment was closely associated with T‐cell signaling pathways, correlating with increased T‐cell infiltration and activation (Figure 5B). KEGG pathway analysis highlighted the involvement of DEGs in tumor and immune microenvironment‐related pathways, such as the TNF pathway and JAK‐STAT signaling pathway (Figure 5C). GO analysis, conducted across three categories (cellular component [CC], biological process [BP], and molecular function [MF]), further confirmed the enrichment of DEGs in anti‐tumor immune‐related pathways, including the JAK‐STAT pathway and CD86 synthesis (Figure 5D–F). GSEA also indicated a strong association between RFA+FSRP treatment and upregulation of the TBK1 pathway (Figure 5G). To explore this further, changes in TBK1 mRNA levels were assessed via RT‐qPCR, which showed that RFA+FSRP did not alter TBK1 mRNA levels (Figure 5H). However, IF staining revealed significant upregulation of TBK1 protein levels following FSRP treatment (Figure 5I,J), suggesting that FSRP modulates TBK1 via post‐translational mechanisms rather than transcriptional regulation. Western blot analysis confirmed that FSRP enhanced the RFA‐induced upregulation of phosphorylated TBK1 (p‐TBK1) and phosphorylated IRF3 (p‐IRF3) proteins in HCC cells (Figure 5K,L). Furthermore, IP assays demonstrated that FSRP promoted TBK1 deubiquitination, thereby stabilizing TBK1 protein levels (Figure 5M,N). These results indicate that FABP5 inhibition triggers activation of the STING/TBK1 pathway by regulating TBK1 protein stability through post‐translational modifications. To validate the mechanistic specificity of STING/TBK1 pathway activation, we performed loss‐of‐function studies using the selective cGAS inhibitor C176. Western blot analysis demonstrated that C176 treatment abolished the hyperthermia/FSRP‐induced enhancement of TBK1/IRF3 phosphorylation (p‐TBK1/p‐IRF3) (Figure S7, Supporting Information). These findings conclusively establish that pathway activation is strictly dependent on upstream cGAS regulation, thereby excluding potential off‐target effects. Extensive evidence supports the notion that activation of the STING/TBK1 pathway significantly enhances anti‐tumor immune responses.^[^ ^38^ ^]^ The results of this study demonstrate that nanocarrier‐mediated FABP5 knockout effectively amplifies the anti‐tumor immune effects of RFA via activation of the STING/TBK1 pathway.

### 2.6 Dual‐Loaded Nanoparticles Targeting Tumor Cell‐Intrinsic FABP5 Combined with RFA Synergistically Inhibit HCC Progression and Distant Metastasis

**Figure 6:**
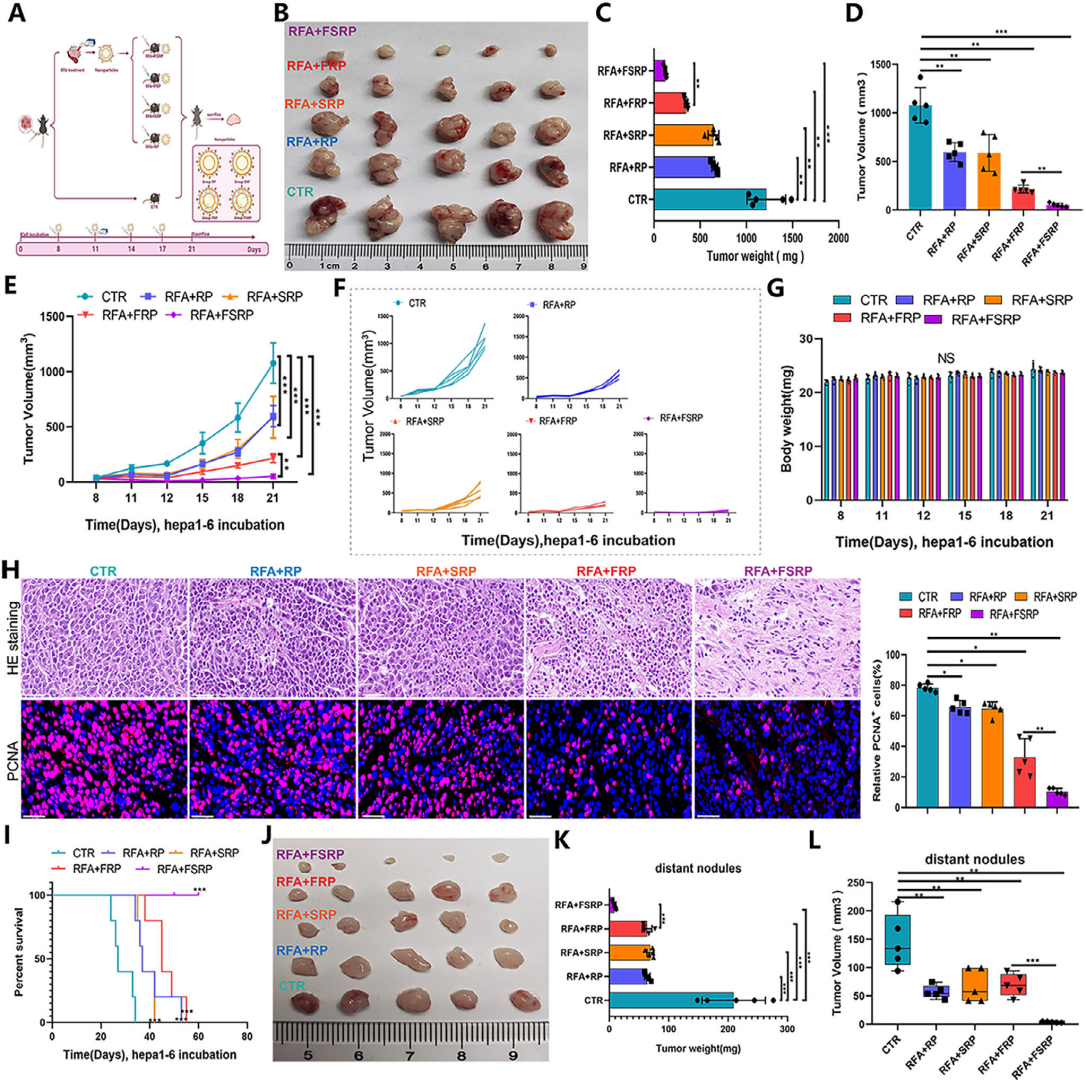
Dual‐loaded nanoparticles targeting FABP5 combined with RFA synergistically inhibit HCC progression and metastasis. A) Flowchart illustrating the mouse model protocol post‐RFA. B) Tumor size comparisons under different treatment conditions following RFA. C,D) Histograms depicting tumor weight and volume statistics for each group. E) Line graph illustrating tumor growth kinetics across treatment groups. F) Individual growth trajectories of mice represented in a line graph. G) Statistical histogram showing weight changes in mice across treatment groups. H) Representative H&E images depicting HCC tumor tissue structure under different conditions, alongside immunofluorescence images of PCNA‐positive cells (PCNA in red, DAPI in blue) with quantified results. I) Survival curve demonstrating the percent survival of Hepa1‐6‐bearing mice under various treatments. J) Tumor size plots shown for each treatment condition. K) Histograms presenting tumor weight statistics. L) Box plots showing tumor volume distributions. Statistical significance, ^*^ *p*< 0.05; ^**^ *p*< 0.01; ^***^ *p*< 0.001.

To further investigate the synergistic anti‐tumor effects of nanoparticles targeting FABP5 combined with RFA in vivo, mice were divided into four groups: RP (control), SRP, FRP, and FSRP, with the latter three serving as treatment groups (**Figure** **6**A). Experimental results revealed that tumor weight and volume were significantly smaller in the treatment groups compared to the control group, with slower tumor growth rates observed. Nanoparticles loaded individually with SPIO or sgFABP5 demonstrated comparable efficacy, while the double‐loaded nanoparticles exhibited the most pronounced tumor inhibition (Figure 6B–F). Histological analysis confirmed no significant structural changes in major tissues across all groups (Figure S6A, Supporting Information). Liver function indicators (ALT, AST, ALB), cardiac enzyme markers (CK), and renal function parameters (CR, BUN) remained stable (Figure S6B–G, Supporting Information), indicating excellent biocompatibility of the nanomaterials. Moreover, no notable alterations in body weight were observed among the groups, and the nanoparticles exhibited negligible cytotoxicity throughout the experiment (Figure 6G). PCNA, a marker of tumor cell proliferation,^[^ ^39^, ^40^ ^]^ was evaluated via IF staining. Both SPIO‐ and sgFABP5‐loaded nanoparticles reduced PCNA expression compared to the control group, with the double‐loaded nanoparticles showing the most significant reduction, highlighting their superior anti‐proliferative effects (Figure 6H). Furthermore, the combination of RFA and double‐loaded nanoparticles significantly improved mouse survival rates (Figure 6I). To further assess the anti‐tumor efficacy of double‐loaded nanoparticles, a metastatic Hepa1‐6 tumor ablation model was established. The results demonstrated that all nanoparticle treatments inhibited tumor growth in terms of size, weight, and volume, with the double‐loaded nanoparticles exerting the most potent anti‐metastatic effect, including a remarkable capacity to eliminate distant metastases (Figure 6J–L). Collectively, these results underscore the potential of the nanoparticle co‐delivery system in enhancing the anti‐tumor efficacy of RFA against HCC.

### 2.7 Nanocarrier Targeting Tumor Cell‐Intrinsic FABP5 Enhances RFA‐Mediated Anti‐Tumor Immune Effects

**Figure 7:**
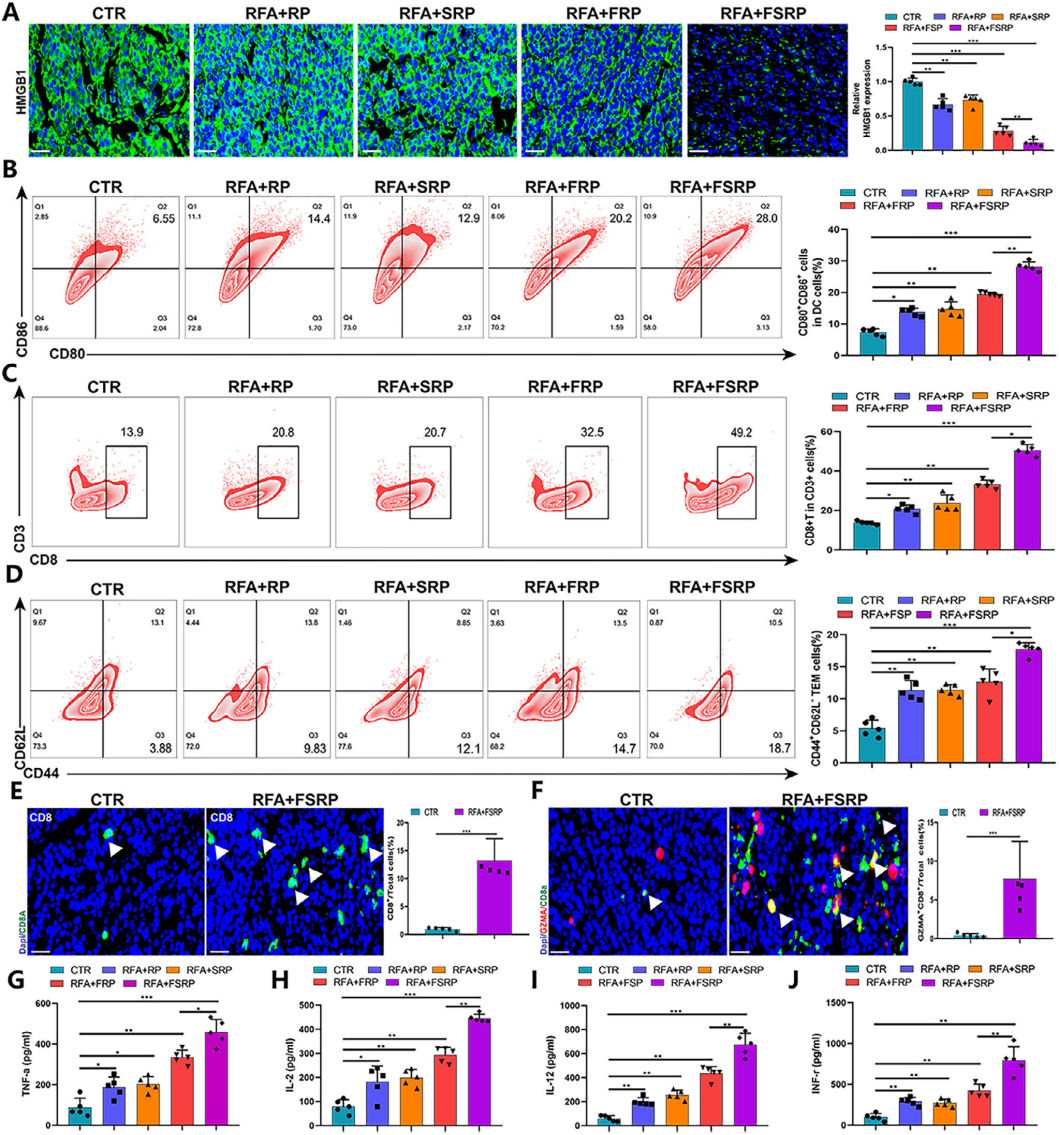
Nanocarrier targeting FABP5 enhances RFA‐induced anti‐tumor immune effects. A) Representative immunofluorescence staining showing relative HMGB1 expression levels in HCC tumor tissues, with histograms quantifying expression levels. B) Flow cytometry false‐color plots depicting CD80 and CD86 expression in live‐cell gated HCC tumor tissues, with histograms showing the percentage of CD80+CD86+ DCs. C) False‐color plots displaying CD8 and CD3 expression, with statistical histograms quantifying the proportion of CD8+ T cells among CD3+ cells. D) Flow cytometry plot highlighting CD44 and CD62L expression, with histograms indicating the percentage of CD44^+^ CD62L^−^ TEM cells. E) Immunofluorescence images displaying CD8+ cells in HCC tumor tissues. F) Representative images showing CD8+ and GZMA+ cells in HCC tumor tissues, alongside the statistical percentage of GZMA+ cells among CD8+ cells. G–J) Levels of TNF‐α (G), IL‐2 (H), IL‐12 (I), and INF‐γ (J) quantified in HCC tumor tissues under different treatment conditions.

The anti‐tumor immune effects of FABP5‐targeting co‐delivery nanoparticles combined with RFA were further examined in HCC. The nanocarrier targeting tumor cell‐intrinsic FABP5 resulted in the downregulation of HMGB1 expression, thereby amplifying the ICD induced by RFA (**Figure** **7**A). Flow cytometry analysis revealed significant increases in CD80+CD86+ DCs (Figure 7B), CD8+CD3+ T cells (Figure 7C), and CD44+CD62L‐TEM cells (Figure 7D), indicating that FABP5 inhibition effectively enhanced immune responses in the tumor microenvironment. IF assays further demonstrated a marked increase in GZMA+CD8+ double‐positive cells and total CD8+ T cells following treatment with the nanocarrier system (Figure 7E,F). Additionally, ELISA results showed elevated levels of RFA‐induced anti‐tumor cytokines, including tumor necrosis factor‐α (TNF‐α), interleukin‐2 (IL‐2), interleukin‐12 (IL‐12), and interferon‐γ (IFN‐γ) (Figure 7G–J). Collectively, these results confirm that FABP5 knockout rewires the tumor immune microenvironment and enhances RFA‐mediated anti‐tumor immune responses in HCC.

### 2.8 Ferroptosis Inhibitor Reverses Anti‐Tumor Effects Induced by Targeting the FABP5 Co‐Delivery System Combined with RFA Treatment in HCC

**Figure 8:**
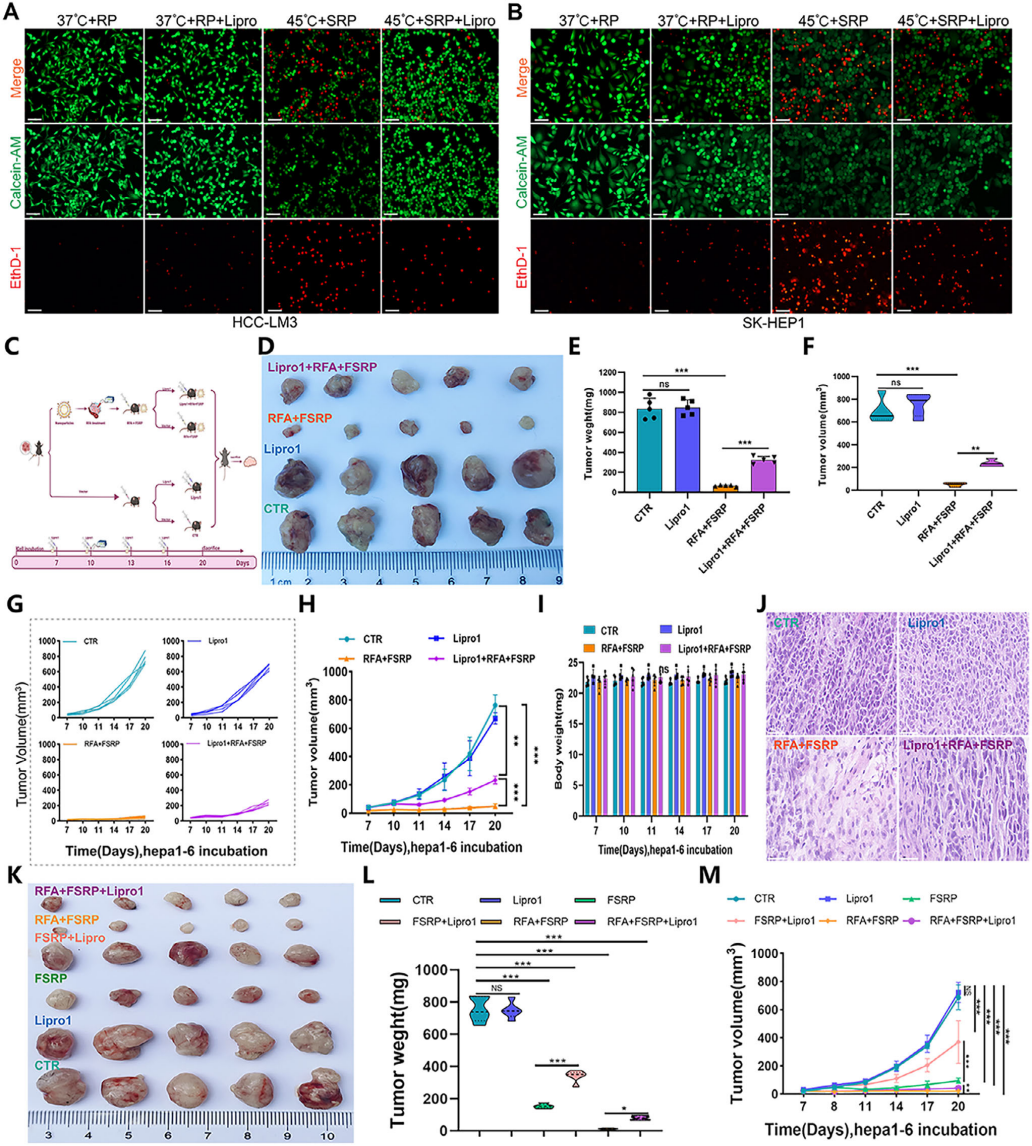
Ferroptosis inhibitor reverses anti‐tumor effects induced by targeting the FABP5 co‐delivery system combined with RFA treatment in HCC A) HCC‐LM3 and B) SK‐HEP1 cells were subjected to the following treatments: 37 °C+RP, 37 °C+RP+Lipro, 45 °C+SRP, and 45 °C+SRP+Lipro. Cell viability was assessed by dual staining with Calcein‐AM (green, viable cells) and EthD‐1 (red, non‐viable cells).C) Schematic flowchart outlining the animal model of HCC cells treated with Lipro1, RFA, and the nanoparticle co‐delivery system. D) Tumor size comparison across treatment groups. E) Histograms presenting tumor weight statistics for each group. F) Violin plots depicting tumor volume distributions across treatment groups. G) Line graph illustrates individual mouse growth trajectories. H) Tumor growth curves shown for each treatment group. I) Histograms displaying weight changes of mice under different treatments. J) Representative H&E images depicting tumor tissue morphology across treatment groups. K) Macroscopic images of resected tumor specimens from experimental groups, control (CTR), Liproxstatin‐1 (Lipro1), FSRP, FSRP+Lipro1, RFA+FSRP, and RFA+FSRP+Lipro1. L) Violin plots depicting tumor volume distributions across treatment groups. M) Temporal progression of tumor volume among experimental groups.

Given the prior evidence linking the anti‐tumor effects of FABP5‐targeting nanoparticles combined with RFA to ferroptosis induction in HCC, this mechanism was further validated using the ferroptosis inhibitor Lipro1. Using a live/dead cell staining assay, the combination of hyperthermic treatment (45 °C) and SRP significantly induced cell death in two hepatocellular carcinoma cell lines (HCC‐LM3 and SK‐HEP‐1), as demonstrated by attenuated fluorescence intensity of viable cell marker (Calcein‐AM, green) and enhanced fluorescence intensity of dead cell marker (EthD‐1, red). Administration of the ferroptosis inhibitor Lipro1 effectively reversed this process, significantly restoring cell viability signals while reducing dead cell signals. Notably, Lipro1 demonstrated no detectable cytotoxicity under physiological temperature conditions (37 °C) (**Figure** **8**A,B). Following FABP5 inhibition via nanocarriers, C11‐BODIPY fluorescence assays revealed a significant increase in lipid peroxidation and a corresponding decrease in GPX4 expression in tumor cells, indicating that FABP5 inhibition enhances RFA‐induced ferroptosis (Figure S8A, Supporting Information). To confirm the role of ferroptosis, Lipro1 was co‐administered with double‐loaded nanoparticles, revealing that Lipro1 significantly reversed the tumor‐suppressive effects of the nanoparticles, as evidenced by increased tumor weight, volume, and growth rate (Figure 8C–H). Liver function indices (ALT, AST, ALB), cardiac myosin levels (CK), and renal function parameters (CR, BUN) in mice showed no significant changes, underscoring the excellent biocompatibility of both Lipro1 and the nanomaterials (Figure S8B,C, Supporting Information). Notably, Lipro1 exhibited no significant biotoxicity (Figure 8I), but its administration markedly inhibited the anti‐tumor effects of sgFABP5, as confirmed by increased PCNA expression in Lipro1‐treated cells relative to the RFA+FSRP group (Figures 8J; S8D, Supporting Information).

To further investigate the antitumor efficacy of combined RFA and FSRP treatment and its ferroptosis‐dependent mechanism in vivo, we comprehensively assessed the therapeutic efficacy of various treatment modalities on tumor progression in tumor‐bearing mice. The results demonstrated that both FSRP monotherapy and combined RFA+FSRP treatment significantly inhibited tumor progression, with the combination therapy demonstrating superior antitumor activity. Notably, the antitumor effects of both therapeutic approaches were effectively attenuated by Lipro1 administration (Figures 8K–M; S8E,F, Supporting Information), while Lipro1 exhibited no appreciable systemic toxicity (Figure S8G, Supporting Information). Our comprehensive investigations revealed that the combination of FABP5‐targeted nanoparticles with RFA exhibited robust antitumor efficacy against HCC via ferroptosis‐dependent mechanisms, which could be specifically attenuated by Lipro1 intervention. Systematic in vitro and in vivo evaluations confirmed the favorable safety profile of this combination therapy and demonstrated the critical involvement of the ferroptotic pathway in mediating its therapeutic effects.

### 2.9 Targeting the FABP5 Co‐Delivery System Combined with RFA and Anti‐PDL1 Effectively Suppressed the Progression of Liver Cancer

**Figure 9:**
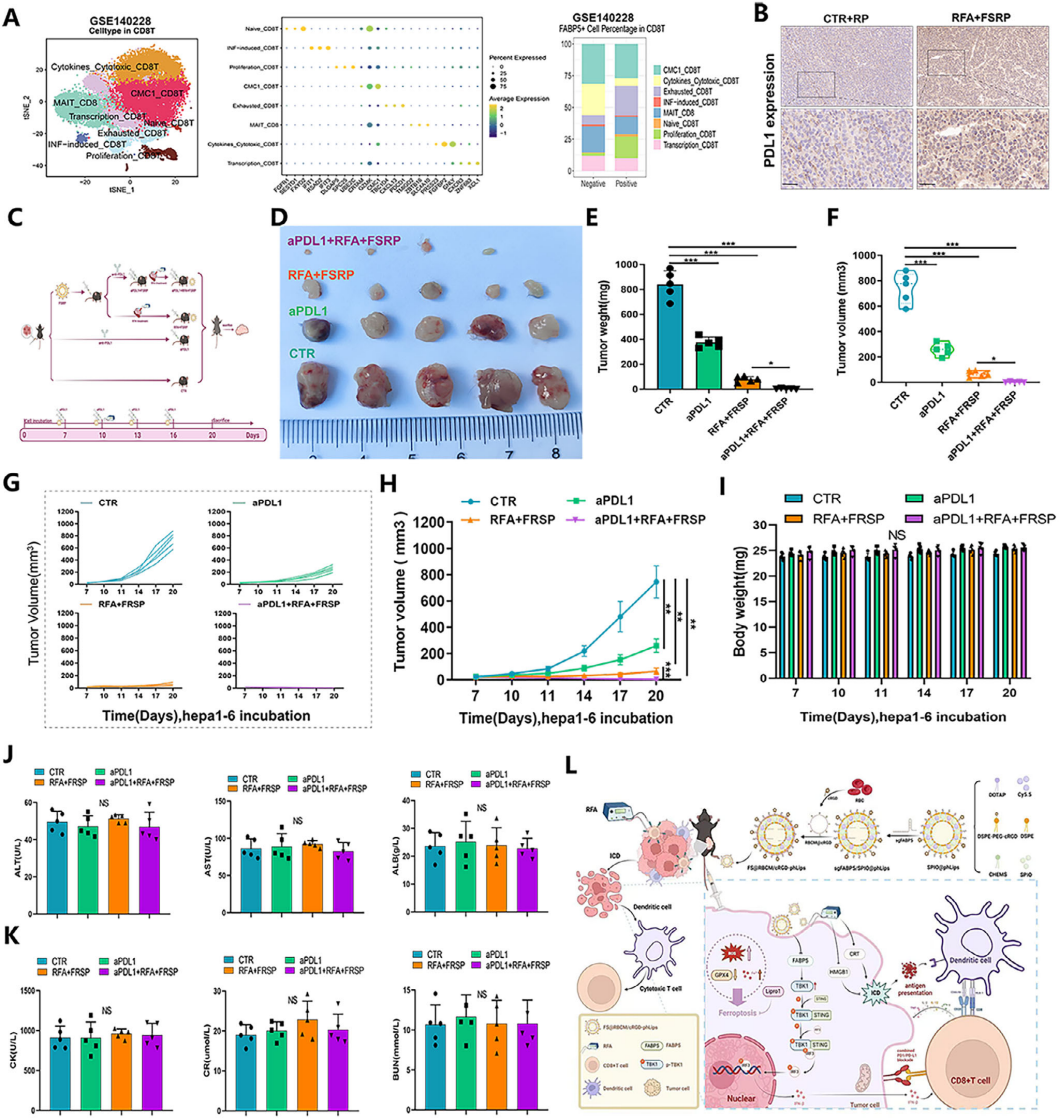
Targeting the FABP5 co‐delivery system combined with RFA and anti‐PD‐L1 therapy effectively suppresses liver cancer progression. A) Comparative analysis of cellular composition reveals that the FABP5‐negative group has a markedly higher proportion of cytotoxic CD8+ T cells in HCC tumor tissues compared to the FABP5‐positive group. B) PD‐L1 expression in HCC tumor tissues was compared between groups treated with or without RFA combined with the dual‐loaded nanoparticle system. C) Schematic representation of therapeutic protocols for the Hepa1‐6 tumor mouse model. D) Tumor size comparisons for different treatment groups. E) Histogram depicting tumor weight statistics across groups. F) Violin plot presenting tumor volume distributions across groups. G) Line graph showing individual mouse growth trajectories under different treatments. H) Tumor growth curves for each group. I) Statistical histograms displaying changes in body weight across treatment groups. J) Blood levels of ALT, AST, and ALB measured under different treatment conditions. K) Levels of CK, creatinine (CR), and blood urea nitrogen (BUN) for each group. L) Mechanistic diagram illustrating the anti‐liver cancer effects of the dual‐loaded nanoparticle co‐delivery system.

Previous findings demonstrated that nanocarrier‐mediated FABP5 knockout enhances the efficacy of immunotherapy. To further validate the anti‐tumor effects of FABP5 knockout combined with RFA in HCC immunotherapy, the influence of FABP5 on the TME was analyzed. CD8+ T cells from the GSE140228 cohort were sorted and subjected to evaluation. A significant reduction in the proportion of proliferative CD8+ T cells was observed in the FABP5‐positive group compared to the FABP5‐negative group, indicating a more robust anti‐tumor immune response in the FABP5‐negative cohort (**Figure** **9**A). These findings align with previous results highlighting the therapeutic benefits of FABP5 downregulation. Immunohistochemistry revealed a pronounced increase in PD‐L1 expression following treatment with the targeted FABP5 co‐delivery system, suggesting that the combination of double‐loaded nanoparticles with RFA could enhance the efficacy of anti‐PD‐L1 immunotherapy in HCC (Figure 9B). Administration of anti‐PD‐L1 antibodies in the tumor model significantly amplified the inhibitory effects of double‐loaded nanoparticles, leading to substantial reductions in tumor weight, volume, and growth rate (Figure 9C–H). No discernible biotoxicity was detected throughout the experimental period (Figure 9I), and liver function parameters (ALT, AST, ALB), cardiac myosin levels (CK), and renal function indicators (CR, BUN) remained stable, confirming the excellent biocompatibility of the treatment (Figure 9J,K). These results demonstrate that targeting tumor cell‐intrinsic FABP5 promotes RFA‐induced ferroptosis and rewires the intratumoral immune landscape by activating the STING/TBK1 pathway and stabilizing TBK1 protein. Moreover, the combination of the FABP5‐targeting nanocarrier co‐delivery system with RFA and anti‐PD‐L1 monoclonal antibodies constitutes a promising and effective therapeutic strategy for HCC.

## 3 Discussion

HCC is the most prevalent primary liver malignancy and is associated with a poor prognosis. Viral hepatitis and alcohol‐induced liver damage are significant etiological factors. Current therapeutic options for HCC remain limited, emphasizing the urgent need for innovative and effective treatment strategies. Among available modalities, RFA is the most widely utilized due to its efficacy, reproducibility, low complication rate, and favorable safety profile. RFA induces tumor cell death by denaturing proteins within local tissue, while also enhancing immune cell infiltration into the TME, thereby exerting a tumoricidal effect.^[^ ^41^ ^]^ This study presents a novel nano‐strategy designed to enhance the therapeutic efficacy of RFA in HCC by targeting FABP5, a critical molecule implicated in HCC progression. Our findings demonstrate an increase in FABP5 expression following RFA treatment, and that reducing FABP5 levels significantly enhances therapeutic outcomes in HCC. To achieve precise suppression of FABP5 in tumor cells and maximize anti‐tumor effects, a targeted nanoparticle system (FS@RBCM/cRGD‐phLips) was developed. This system incorporates sgFABP5 and SPIO to deliver targeted therapy. When combined with RFA, these nanoparticles exhibited a synergistic anti‐tumor effect. Specifically, the delivery of sgFABP5 significantly augmented the tumor‐killing efficiency of RFA. Mechanistic studies revealed that nanocarrier‐mediated FABP5 knockout promoted RFA‐induced ferroptosis by elevating iron ion accumulation and lipid peroxidation, processes that drive tumor cell death and suppress proliferation.

Additionally, from a metabolic perspective, FABP5 functions as a critical mediator of tumor progression. FABP5 orchestrates pro‐proliferative, survival, and invasive signaling cascades within tumor cells, while concurrently modulating the immunosuppressive tumor microenvironment through perturbation of immune cell lipid metabolism. Therefore, genetic targeting of FABP5 not only directly suppresses the malignant phenotype of tumor cells but, more importantly, specifically reverses FABP5‐mediated immune metabolic dysregulation. Specifically, FABP5 inhibition attenuates macrophage uptake and transport of tumor‐derived unsaturated fatty acids, suppresses PPARγ activity, and consequently downregulates immune checkpoint molecules, including PD‐L1, CD47.^[^ ^42^ ^]^ Notably, FABP5 knockout further alleviates T cell lipid uptake impediments, thereby preserving their cytotoxic function.^[^ ^43^ ^]^Compared with existing nano‐therapeutic strategies for enhancing RFA efficacy, including the recently reported MFMP nanodrug^[^ ^44^ ^]^ and P@Fe SAZ nanozyme,^[^ ^45^ ^]^ our system achieves selective targeting of post‐RFA overexpressed FABP5, thus effectively inhibiting the critical immune escape pathway in HCC. Moreover, our system provides real‐time monitoring of nanoparticle biodistribution and therapeutic response in vivo. These findings provide compelling evidence that the combination of FABP5‐targeting nanoparticles and RFA constitutes an effective therapeutic strategy. By inducing and amplifying ferroptosis activity, this approach holds the potential to significantly improve the clinical outcomes of HCC treatment.

The results further demonstrated that nanocarriers targeting FABP5 effectively remodeled the anti‐tumor immune response in HCC. Enhanced infiltration of immune cells, including mature DCs and cytotoxic T cells, was observed, accompanied by a notable increase in PD‐L1 expression under FABP5 deficiency treatment. Importantly, the inhibition of FABP5 amplified and sustained the anti‐tumor immune response induced by RFA, with the activation of the STING/TBK1 pathway playing a critical role. Besides the STING/TBK1 pathway revealed in this study, recent research has shown that factors such as ADAM9 can modulate the HCC immune microenvironment through regulation of ferroptosis.^[^ ^46^ ^]^While previous studies have demonstrated that hyperactivation of this pathway may induce benign liver injury through pro‐inflammatory cytokine release,^[^ ^47^ ^]^ the pathway activation observed in our study is maintained within therapeutic windows, with its critical contribution to antitumor immunity significantly exceeding potential adverse effects. Furthermore, nanocarrier‐mediated FABP5 deficiency facilitated RFA‐induced ICD, enhancing the antigen‐presenting capacity of DCs and promoting T‐cell infiltration. This dual effect not only strengthened the immune response but also minimized the escape of residual tumor cells in HCC.

In recent years, immunotherapy has emerged as a transformative clinical strategy for cancer treatment.^[^ ^48^ ^]^ Tumor cells often upregulate PD‐L1 expression, exploiting the PD‐L1/PD‐1 signaling axis to evade T cell‐mediated immune responses.^[^ ^49^, ^50^ ^]^ Blockade of the PD‐L1/PD‐1 pathway has been shown to elicit robust anti‐tumor effects in patients with advanced cancers,^[^ ^51^, ^52^, ^53^ ^]^ establishing this approach as a cornerstone for developing immune checkpoint blockade (ICB) therapies and combination regimens. However, despite its promise, PD‐L1‐targeted immunotherapy faces significant challenges, necessitating innovative strategies to enhance its efficacy. The integration of nanoparticles with PD‐L1 inhibition offers a novel avenue for advancing cancer therapy. In this study, the therapeutic efficacy of dual‐loaded nanoparticles combined with RFA and PD‐L1 inhibitors was evaluated using in vivo experimental models. The findings revealed that PD‐L1 inhibition significantly augmented the anti‐tumor effects of the dual‐loaded nanoparticle and RFA combination. This strategy leverages the synergistic effects of FABP5 targeting, RFA, and immune checkpoint blockade to achieve enhanced tumor suppression.

The combination of RFA and PD‐L1 inhibitors based on a FABP5‐targeting dual‐loaded nanoparticle delivery system represents a groundbreaking approach for HCC treatment. This strategy offers a promising pathway to significantly improve therapeutic efficacy and holds substantial potential for clinical application in cancer therapy.

While this FABP5‐targeted nanosystem has demonstrated promising efficacy in preclinical models, its clinical translation necessitates systematic evaluation of critical challenges. The primary safety consideration concerns potential off‐target immune effects, although immunogenicity of the CRISPR components has been reduced through sgRNA optimization^[^ ^54^ ^]^ and erythrocyte membrane camouflage,^[^ ^55^ ^]^ comprehensive validation of gene editing specificity in normal liver tissue and circulating immune cells remains essential through large animal models. From a manufacturing perspective, production processes for the system's core components (degradable lipid shell, SPIO, CRISPR ribonucleoprotein complex) can be implemented using established GMP processes validated for approved LNP‐based drugs.^[^ ^56^ ^]^ Proposed clinical trials should evaluate two key aspects, 1) Vector‐related immunotoxicity,^[^ ^57^ ^]^ encompassing systemic inflammatory responses (serum IL‐6/TNF‐α levels) and organ‐specific immune infiltration (particularly liver/spleen histopathology); 2) Targeted delivery efficiency, assessed through intraoperative real‐time MRI monitoring of intratumoral SPIO distribution (ΔR_2_ values),^[^ ^58^ ^]^ coupled with post‐procedural biopsy validation of tumor cell editing efficiency (T7E1 assay and NGS off‐target analysis).^[^ ^59^ ^]^

## 4 Conclusion 

In conclusion, this study identifies FABP5 as a promising therapeutic target for HCC, with a strong association with the efficacy of RFA. An innovative dual‐loaded nanoparticle system (FS@RBCM/cRGD‐phLips) was developed, incorporating SPIO into sgFABP5 to enhance the targeting capability of the nanoparticle and significantly amplify the tumor‐destructive effects of RFA. Nanocarrier‐mediated FABP5 deletion was found to promote RFA‐induced ferroptosis and anti‐tumor immune responses, including increased infiltration of CD8+ T cells and effector memory T cells, through activation of the STING/TBK1 pathway and stabilization of TBK1 protein. Moreover, the combination of the FABP5‐targeting nanocarrier co‐delivery system with RFA and PD‐L1 monoclonal antibodies demonstrated exceptional efficacy as a therapeutic strategy against HCC. This innovative approach, which integrates gene editing technology, nanotechnology, and immunotherapy, not only provides a novel and effective framework for cancer treatment but also holds significant potential for clinical application.

## 5 Experimental Section

### Differentially Expressed Gene (DEG) and Enrichment Analyses

The mRNA sequencing data and clinical information of patients with HCC were obtained from the TCGA, ICGC, and GEO databases. Patients with incomplete follow‐up data were excluded from the analysis. Fresh HCC‐LM3 tissue samples were collected from the RFA+FSRP group (experimental group) and the RFA+RP group (control group), followed by mRNA extraction. The cDNA library was synthesized after mRNA fragmentation, and large‐scale sequencing was performed. Rigorous quality control was applied to the sequencing results to generate raw sequencing data files. DEGs were identified using the limma package in R, based on the criteria of an adjusted *p*‐value < 0.05 and |log fold change (logFC)| >1. Gene Ontology (GO) and Kyoto Encyclopedia of Genes and Genomes (KEGG) enrichment analyses were conducted to identify DEGs and investigate functional and regulatory signaling pathways associated with RFA (adjusted *p*< 0.05).

### Cell Culture and Cell Hyperthermia Administration

The study utilized the Hepa1‐6, SK‐HEP1, and HCC‐LM3 cell lines, procured from the American Type Culture Collection (ATCC) and validated through short tandem repeat analysis. SK‐HEP1, HCC‐LM3, and Hepa1‐6 cells were cultured in the DMEM complete medium, while mouse bone marrow‐derived dendritic cells (DCs) were cultured in the RPMI‐1640 complete medium. Serum inactivation for the DC cultures was performed at 56 °C for 30 min. All cell lines were maintained in a CO_2_ incubator at 37 °C. To simulate thermal ablation in vitro, SK‐HEP1 and HCC‐LM3 cells were subjected to heat treatment in a water bath at 45 °C for 10 min.

### Immunofluorescence (IF) and Immunohistochemistry (IHC)

Fixed tissues were dehydrated sequentially in graded alcohol concentrations, transitioning from lower to higher levels. Following dehydration, the tissues were embedded in melted paraffin wax until fully infiltrated. Solidified paraffin blocks were sectioned into slices, which were then dried in an oven at 60 °C for 1 h. The dried slices were immersed in dimethyl acetate and subsequently deparaffinized using xylene.

For IF, paraffin sections were deparaffinized, rehydrated, and subjected to antigen retrieval. Membrane permeabilization was achieved using PBS containing 0.3% Triton X‐100 for 30 min. Blocking was performed with a sealing solution at room temperature for 1 h, after which primary antibodies were applied and incubated overnight at 4 °C. The sections were brought to room temperature and washed five times with PBS. Secondary antibodies were then added and incubated at room temperature for 1 h, followed by three additional washes with PBS. DAPI staining was performed by incubating the sections in DAPI solution for 10 min at room temperature, followed by three final PBS washes. The slices were sealed with an anti‐quenching mounting medium and left at room temperature for 24 h to ensure complete stabilization.

For IHC, tissue sections were dried in an oven at 60 °C for 1 h and deparaffinized twice using xylene. Rehydration was performed with 90%, 80%, and 70% alcohol solutions. The sections were rinsed with TBST and PBS three times each. Endogenous peroxidase activity was blocked using 3% hydrogen peroxide solution for 30 min. Antigen retrieval was conducted with sodium citrate buffer at 100 °C, followed by three PBS washes. The sections were blocked with 5% goat serum at room temperature for 1 h, and primary antibodies were applied and incubated overnight at 4 °C. Following primary antibody incubation, the sections were washed with PBS and incubated with secondary antibodies for 1 h, followed by two washes with PBS. DAB chromogen solution was then applied for visualization, and the sections were washed three times with PBS. Hematoxylin staining was conducted for 5 min, followed by washing with distilled water and dehydration through graded alcohol solutions. Neutral resin was used to seal the sections, which were air‐dried and subsequently examined under a microscope, with immunohistochemical images captured for analysis.

### Live/Dead Cell Staining Assay

A mixture of Calcein‐AM and PI was prepared in FBS‐free DMEM medium at a ratio of 20 µl Calcein‐AM to 5 µl PI solution per 10 mL of medium. Cells were washed with PBS at 37 °C before adding 1 mL of the Calcein‐AM/PI solution to each well. The culture dishes were incubated in a CO_2_ incubator at 37 °C for 10–15 min, followed by 3–5 washes with PBS at 37 °C. Subsequently, 1 mL of complete DMEM medium was added, and the cells were observed under a fluorescence microscope.

### Flow Cytometry

Digested Hepa1‐6 tumor cells were resuspended in PBS, and the cell concentration was adjusted to ≈1 × 10^6^ cells mL^−1^ by adding PBS after counting. A volume of 0.5 µl of flow cytometry antibody was added to each 1.5 mL EP tube, and the tubes were incubated in a light‐protected area for 15 min with intermittent shaking (3–5 times) to ensure sufficient mixing of antibody and cells. The tubes were centrifuged at 800 rpm for 10 min at 4 °C, and the cells were washed 2–3 times with pre‐cooled PBS, avoiding light exposure. Flow cytometry was performed using a blank control for gating, voltage adjustment, and de‐adhesion settings. The data were analyzed using FlowJo software. Details of the antibodies used are provided in the Supporting Information.

### Western Blot

For protein analysis, lysates were extracted from HCC‐LM3 and SK‐HEP1 cells using 1% RIPA lysis buffer, and total protein was collected from the supernatant. Protein concentrations were quantified using the BCA method. Proteins were separated via sodium dodecyl sulfate‐polyacrylamide gel electrophoresis (SDS‐PAGE) and transferred onto a polyvinylidene fluoride (PVDF) membrane. The membrane was blocked with TBST containing 5% skimmed milk for 90 min at room temperature and incubated with primary antibodies overnight at 4 °C. After rinsing with TBST, the membrane was incubated with secondary antibodies for 90 min at room temperature, followed by further washes with TBST. Chemiluminescent imaging of protein bands was performed in a dark room using an ECL substrate and a chemiluminescent imaging system. Details of the antibodies used are included in the Supporting Information.

### RNA Extraction and Quantitative Real‐Time PCR (qPCR)

RNA was extracted using the RNA Rapid Extraction Kit from EZBioscience in accordance with the manufacturer's protocol. A total of 1 µg of RNA was reverse‐transcribed using the Reverse Transcription Kit to generate cDNA, which was either immediately used for fluorescence quantitative PCR or stored at −20 °C for future use. The qPCR reaction mixture (10 µL) was prepared with 5 µL of SYBR‐Green fluorescent probe, 1 µL of upstream and downstream primers each, 3 µL of nuclease‐free H_2_O, and 1 µL of cDNA. Real‐time fluorescence qPCR was performed, and the results were analyzed accordingly. Details of the primers utilized in this study are included in the Supporting Information.

### Cell Proliferation/Toxicity

Human LM3, SK‐HEP1, and mouse Hepa1‐6 cells were cultured and digested with trypsin prior to seeding into 96‐well plates at a density of 5000 cells per well in a 200 µL cell suspension. The cells were cultured for 24 h as a control group. Subsequently, @phLips, @RBCM/cRGD‐phLips, and SPIO@RBCM/cRGD‐phLips were introduced into the culture medium and incubated for an additional 24 h. After the cells adhered to the plate, the medium was replaced with a DMEM complete medium containing 10% CCK8 solution, and the cells were incubated for 2–4 h. The plates were then placed into a zymography instrument, which was set to oscillate for 30 seconds, and the absorbance at 450 nm was measured.

### Animal Models

Subcutaneous tumor‐bearing models and an in situ transplantation tumor model were established using Hepa1‐6 hepatocellular carcinoma cells in mice. A total of 200 µL of Hepa1‐6 cell suspension at a concentration of 1 × 10^6^ cells mL^−1^ was injected subcutaneously into the inner thigh of each mouse. When the tumor volume reached ≈100 mm^3^, an 18G bipolar ablation needle was inserted into the tumor tissue. The radiofrequency tail pin was connected to a radiofrequency transmitter, and the ablation was performed at 60 °C for a duration of 2 min.

The weight of the mice was recorded every three days, and tumor dimensions (length [L] and width [W]) were measured using vernier calipers. Tumor volume was calculated using the formula (V) = (L × W^2^)/ 2. Once the tumor volume reached ≈1500 mm^3^, tumors were excised. A portion of the tumor tissue was fixed in 4% paraformaldehyde for histological analysis, while the remaining tissue was stored at −80 °C for further experimental studies.

### Nanomaterials Preparation

Lipid nanoparticles (LNPs) are nanoscale carrier systems constructed from lipid‐based materials. These systems leverage the self‐assembly properties of lipid molecules under specific conditions to form nanoscale vesicular structures capable of encapsulating and protecting drug molecules while facilitating targeted in vivo delivery. In this study, sgRNA and SPIO were encapsulated within LNPs. Transmission electron microscopy (TEM) was employed to assess the LNPs' quality, including size and morphology. The LNPs were administered via tail vein injection.

### Enzyme‐Linked Immunosorbent Assays (ELISA) & Multiplex Cytokine Array Assay

Levels of TNF‐α and INF‐γ in tissue lysates from Hepa1‐6 hepatocellular carcinoma cells in mice were quantified using an ELISA kit (Invitrogen/Thermo Fisher Scientific) following the manufacturer's protocol. Cytokine array kits were obtained from R&D Systems. For the ELISA procedure, antibodies were diluted in 0.05 m pH 9 carbonate encapsulation buffer, and 0.1 mL was added to the reaction wells of polystyrene plates, incubated overnight at 4 °C. The following day, 0.1 mL of appropriately diluted samples was added to the coated wells and incubated at 37 °C for 1 h. Enzyme‐labeled antibodies were then applied to each reaction well. Subsequently, 0.1 mL of freshly prepared TMB substrate solution and 0.05 mL of 2 m sulfuric acid were added. The optical density (OD) of each well was measured at 450 nm using an ELISA reader (410 nm if ABTS was used for color development), with results considered positive if the OD value exceeded 2.1 times that of the negative control.

### Co‐IP Assay

Liposomal nanoparticles were lysed in immunoprecipitation (IP) buffer containing 20 mM Tris‐HCl (pH 8.0), 150 mM NaCl, 2 mM EDTA, and 1% Nonidet P‐40. The lysates were incubated with beads pre‐loaded with liposomal nanoparticles overnight at 4 °C. The beads were collected, and fractions of the bead volume were resolved using SDS‐PAGE, followed by silver staining. Western blot analysis was subsequently performed to assess protein expression levels.

### Statistical Analysis

All experiments were conducted in three independent replicates. Statistical analysis was performed using GraphPad Prism (version 8.0) software and the R package. Quantitative results were expressed as mean ± standard error of mean (SEM). Comparisons between two groups were analyzed using Students *t*‐test for normally distributed data, while non‐normally distributed data were analyzed using the rank‐sum test. For comparisons among multiple groups, one‐way analysis of variance (ANOVA) followed by Tukey's multiple comparisons test was performed. Pearson correlation analysis was used to calculate the R and P values for scatter plots. Statistical significance was defined as *p*<0.05 and denoted as ns (not significant), ^*^ *p*<0.05, ^**^ *p*<0.01, ^***^ *p*<0.001, and ^****^ *p*<0.0001 in figures and figure legends.

## Conflict of Interest

The authors declare no conflict of interest.

## Author Contributions

B.T., X.Z., and Y.S. contributed equally to this work. B.T. and X.Z. performed methodology, investigation, and visualization, and wrote the original draft. Y.S. performed methodology, formal analysis, and visualization. J.L., C.Z., and Z.W. performed validation and formal analysis. S.F. and L.Z. performed software programming and visualization. Y.X. and Q.W. performed resources. Z.Z. performed, resources, and funded acquisition. R.Q., Y.Y., and M.X. performed data curation. J.T. and M.C. performed project administration, wrote, reviewed, and edited the final manuscript. J.J. performed conceptualization, supervision, project administration, and funded acquisition. All authors reviewed the manuscript.

## Consent for Publication

All of the authors are aware of and agree to the content of the paper and their being listed as co‐authors of the paper.

## Supporting information



Supporting Information

## Data Availability

The data that support the findings of this study are available from the corresponding author upon reasonable request.
