# Supplementary material for: Tumor‐Targeted FABP5/STING Cascade Promote Radiofrequency Ablation Induced Ferroptosis and Intratumoral Immune Rewiring in Hepatocellular Carcinoma
Source: Adv Sci (Weinh). 2025 Sep 16;12(45):e07864. doi: 10.1002/advs.202507864 (PMC12677646; doi:10.1002/advs.202507864)
Supplement: Supplementary file 1 — Supporting Information [file ADVS-12-e07864-s001.docx]

**Supporting Information Figures:**


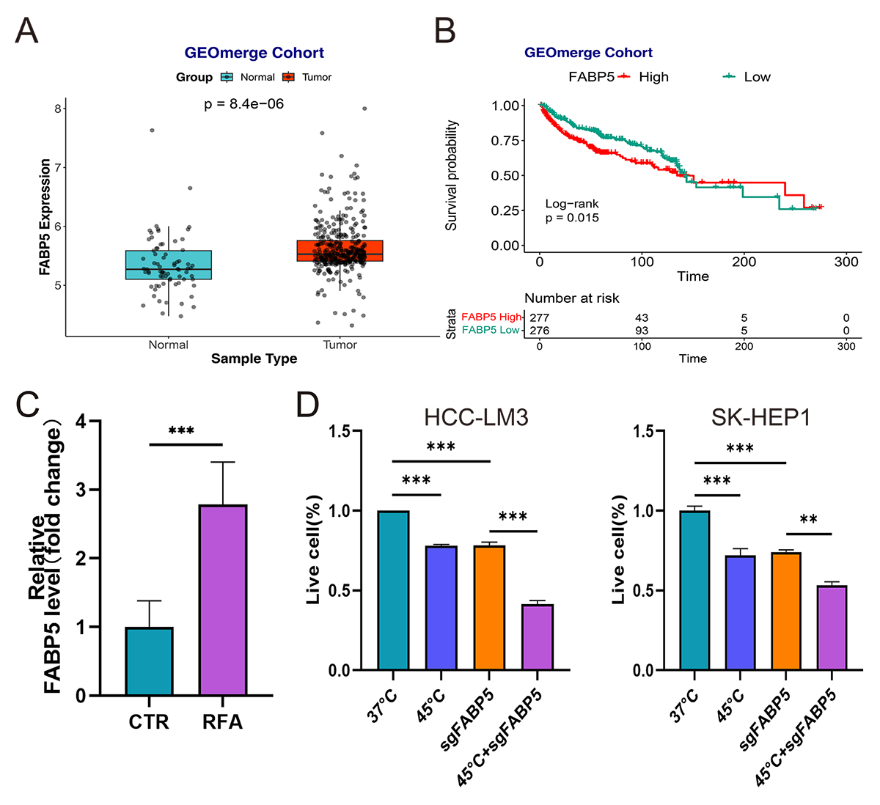


Figure S1. FABP5 is highly expressed in HCC tissues and correlates with RFA treatment
(A) Box plots displaying FABP5 expression levels in normal and tumor samples from the GEOmerge cohort. (B) Kaplan-Meier survival analysis stratifying patients with HCC into high and low FABP5 expression groups. (C) Statistical histogram showing changes in FABP5 levels in Hepa1-6 tumor tissues following RFA treatment. (D) Statistical histogram presenting the percentage of live cells under different treatments in HCC-LM3 and SK-HEP1 cells.


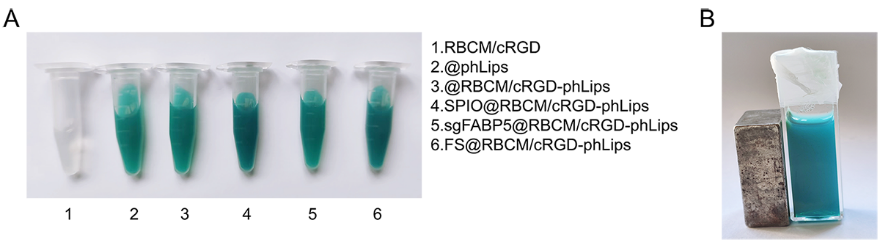


Figure S2. (A) Representative morphological features of the nanoparticle suspension. (B) Representative morphological features of magnetic adsorption nanoparticles.


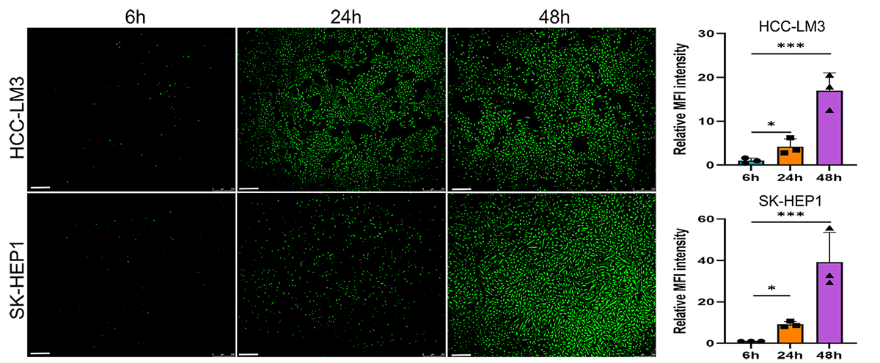


Figure S3. Successful loading of sgRNA plasmid into nanoparticles. Representative IF micrographs of HCC-LM3 and SK-HEP1 cells, accompanied by a histogram illustrating relative GFP fluorescence intensity.


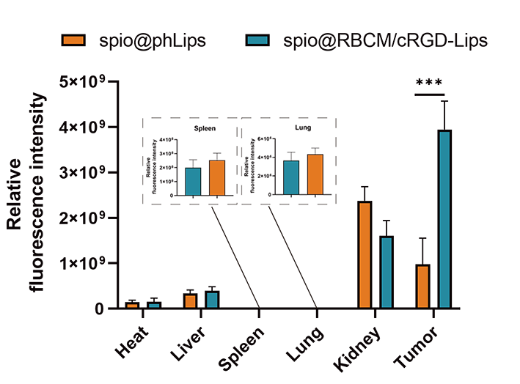


Figure S4. Quantitative analysis of nanoparticle distribution across different organs in the spio@phLips group and spio@RBCM/cRGD-Lips group.


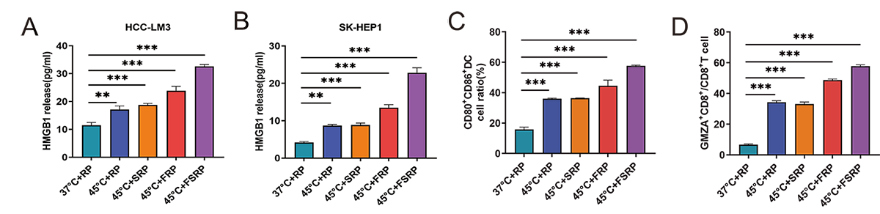


Figure S5. The targeted FABP5 co-delivery system significantly enhances RFA-induced ICD and activates DC-mediated immune responses. (A-B) Histograms depicting HMGB1 release in HCC-LM3 and SK-HEP1 cells under various treatments. (C) Histogram showing the ratio of CD80+CD86+ DC cells in response to different treatments. (D) Histogram of GMZA+CD8+/CD8+ T cells following different treatments.


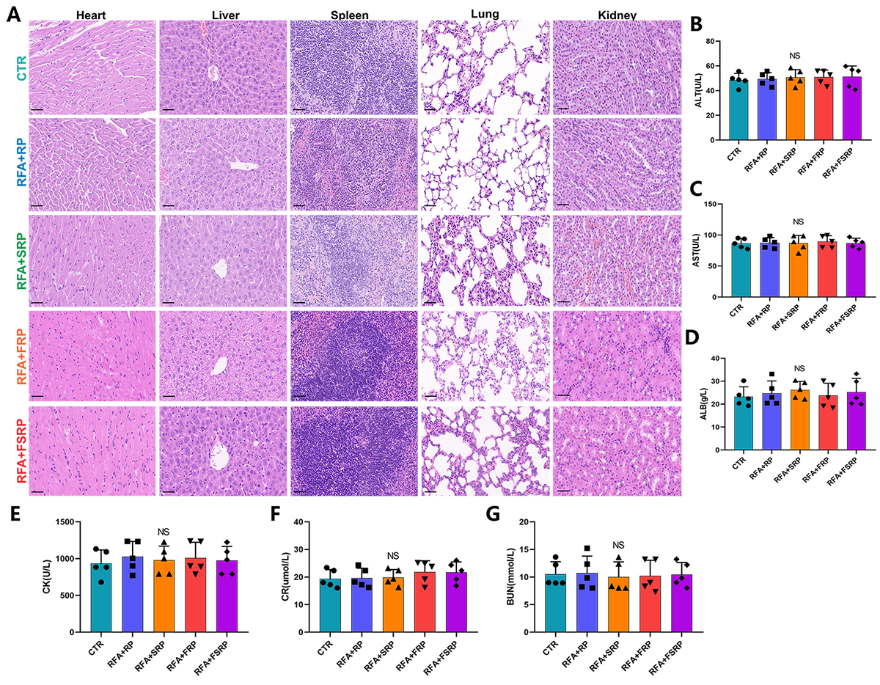


Figure S6. *In vivo* toxicity assessment of various nanoparticle groups. (A) Representative HE staining images showing tissue structure of the heart, liver, spleen, lungs, and kidneys following different treatments. (B-G) Measurement of ALT, CK, BUN, AST, ALB, and CR levels in mice after different treatments.


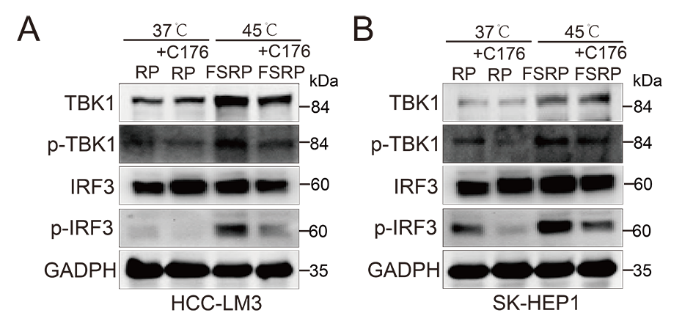


Figure S7. cGAS inhibitor C176 attenuates TBK1-IRF3 phosphorylation co-induced by FSRP and heat treatment. Western blot analysis of TBK1, phosphorylated TBK1 (p-TBK1), IRF3, and phosphorylated IRF3 (p-IRF3) expression in (A) HCC-LM3 and (B) SK-HEP1 cells treated with RP (control) or FSRP (37°C or 45°C, 24 h) in the presence of cGAS inhibitor C176.


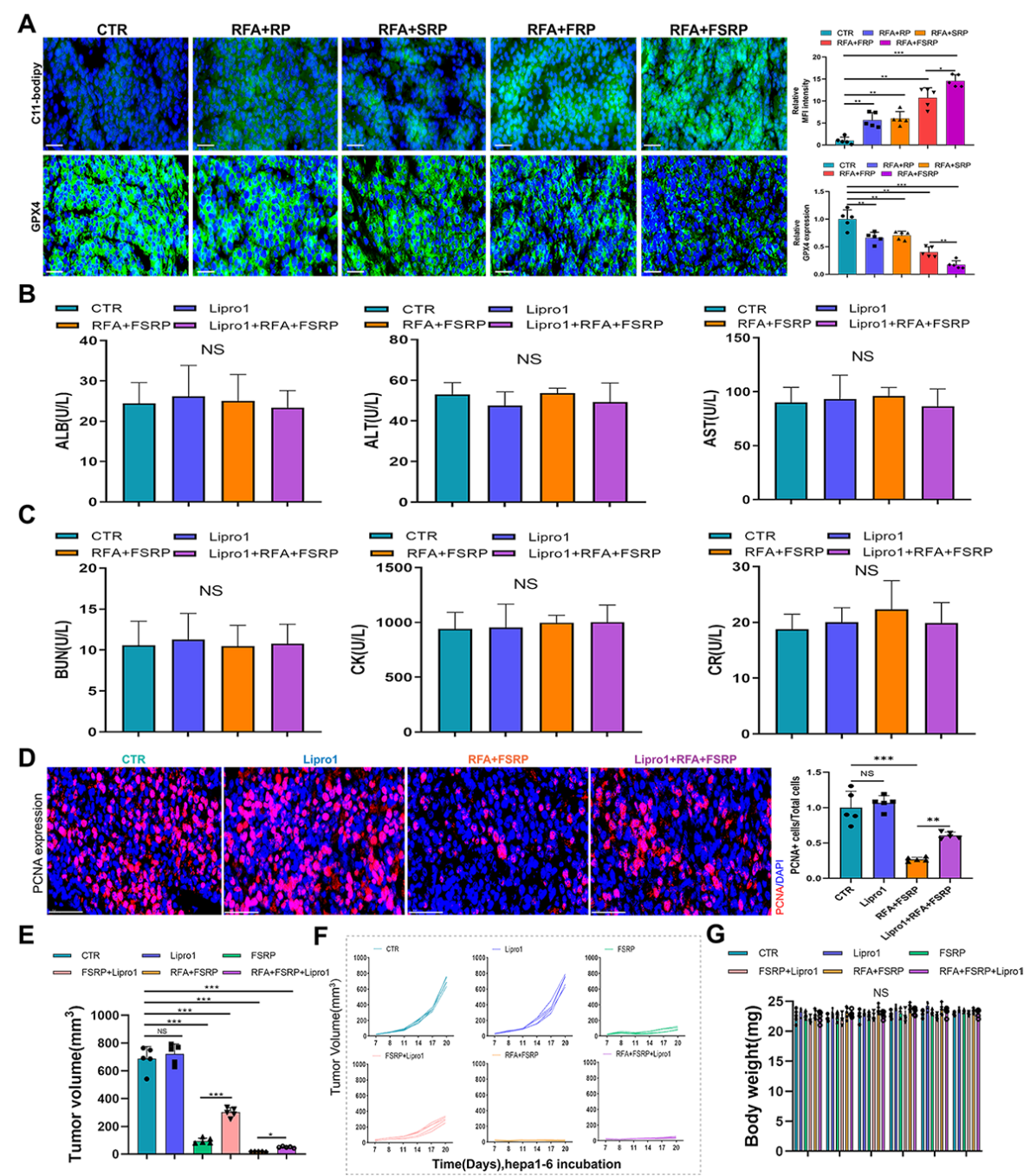


Figure S8. Comprehensive assessment of combined targeted nanoparticle and RFA therapy: antitumor efficacy, ferroptosis mechanism, and systemic safety. (A) Representative C11-BODIPY staining illustrating lipid peroxidation in HCC tumor tissues. Histograms quantifying relative MFI intensity and GPX4 expression in tumor tissues. (B) Blood levels of albumin (ALB), alanine aminotransferase (ALT), and glutamate aminotransferase (AST) in mice across different treatment groups. (C) Measurements of urea nitrogen (BUN), creatine kinase (CK), and creatinine (CR) levels under varying treatment conditions. (D) PCNA expression shown under different treatment conditions, with statistical histograms quantifying the percentage of PCNA-positive cells. (E) Tumor volumes at the experimental endpoint across experimental groups. (F) Temporal changes in tumor volume (days) in the Hepa1-6 tumor-bearing model. (G) Body weight progression (mg) among experimental groups.
